# PET imaging enables direct evaluation of ferroptosis in HCC synergistically induced by anti-PD-L1 and Olaparib

**DOI:** 10.1038/s12276-026-01860-7

**Published:** 2026-10-09

**Authors:** Feng Xiong, Cheng Xu, Pei Wang, Ni Li, Yilin Yang, Buchuan Zhang, Kijung Kwak, Shuang Song, Pengtao You, Xiaohua Zhu, Dalong Ni, Bo Yu

**Affiliations:** 1 Department of Nuclear Medicine, Tongji Hospital, Tongji Medical College, Huazhong University of Science and Technology, Wuhan, China; 2 Department of Nuclear Medicine, Tongji Hospital, Tongji Medical College and State Key Laboratory for Diagnosis and Treatment of Severe Zoonotic Infectious Diseases, Huazhong University of Science and Technology, Wuhan, China; 3 Hubei University of Chinese Medicine, Wuhan, Hubei Province China; 4 Hubei Key Laboratory of Resources and Chemistry of Chinese Medicine, Hubei University of Chinese Medicine, Wuhan, Hubei Province China; 5 Department of Orthopaedics, Shanghai Key Laboratory for Prevention and Treatment of Bone and Joint Diseases, Shanghai Institute of Traumatology and Orthopaedics, Ruijin Hospital, Shanghai Jiao Tong University School of Medicine, Shanghai, China; 6 University of Maryland School of Medicine, Baltimore, MD USA; 7 Suzhou Institute of Biomedical Engineering and Technology, Chinese Academy of Science, Suzhou, China

**Keywords:** Drug development, Molecular imaging

## Abstract

The efficacy of PET image-guided immune checkpoint inhibitor-based therapy in hepatocellular carcinoma (HCC) management is limited by the lack of direct monitoring of therapeutic response. A key challenge is the mismatch between the therapeutic pathway and the imaging target, which hinders the accurate assessment of treatment outcomes. We addressed this challenge by combining anti-PD-L1 immunotherapy with Olaparib, a strategy that promotes ferroptosis in BRCA wild-type HCC by suppressing SLC7A11, thus allowing for therapeutic response evaluation via [^18^F]FASu PET imaging of SLC7A11 expression. The combination treatment resulted in a 57% reduction in tumor volume, accompanied by decreased Ki-67 expression and increased levels of γ-H2AX and 8-OHdG. [^18^F]FASu PET imaging revealed significantly lower SUVmax and tumor-to-background ratios in the combination group, correlating with SLC7A11 downregulation. Mechanistically, GPX4 downregulation, glutathione (GSH) depletion and elevated malondialdehyde (MDA) levels confirmed the activation of ferroptosis. Notably, no therapeutic or imaging response was observed in immunodeficient models. Thus, the SLC7A11-targeted PET tracer [^18^F]FASu offers a clinically translatable biomarker for monitoring treatment efficacy, establishing a novel theragnostic paradigm that directly evaluates treatment response via PET imaging.

## SLC7A11-targeted PET monitors immunotherapy response in hepatocellular carcinoma

Immune checkpoint inhibitor-based combination therapies have improved outcomes in hepatocellular carcinoma, but evaluating treatment response remains challenging because current molecular imaging targets are poorly aligned with therapeutic mechanisms. Here, the authors demonstrate that anti-PD-L1 and the PARP inhibitor Olaparib cooperatively suppress SLC7A11 to induce ferroptosis, enabling direct visualization of therapy-induced molecular changes using SLC7A11-targeted [^18^F]FASu PET. In mouse models, the combination therapy enhanced antitumor immunity, promoted ferroptosis, and markedly reduced tumor growth, whereas decreased [^18^F]FASu uptake accurately reflected SLC7A11 downregulation. These findings establish a therapeutic–imaging target-matched strategy that enables mechanism-based monitoring of immunotherapy responses and supports the development of precision imaging biomarkers for hepatocellular carcinoma.

This summary was initially drafted using artificial intelligence, then revised and fact-checked by the author.

## Introduction

Hepatocellular carcinoma (HCC) ranks as the third leading cause of cancer-related mortality, with a dismal 5-year survival rate^1^. Although immune checkpoint inhibitor (ICI)-based immunotherapies, such as PD-1, PD-L1 and cytotoxic T lymphocyte-associated antigen 4 (CTLA-4) antibodies treatment, have revolutionized the clinical outcome of HCC, only ~15–20% of patients with HCC benefit from a durable response, as well as confront the risk of immunotherapy-related adverse events^2,3^. Thus, image-guided theragnosis is increasingly important for HCC management. However, the controversy on the association of tumor size with response or progression during immunotherapy challenges current image-guided theragnosis^4^. There is a growing consensus that PET imaging is critical in evaluating therapeutic response^5^. Therefore, exploring rational PET image-guided combination therapy is expected to improve ICI-based HCC management.

Reviewing the current paradigm of PET imaging in cancer management reveals several modalities (Supplementary Tables 1 and 2), including the evaluation of cancer cell viability ([^18^F]FDG) and the abundance of lesion targets (immunoPET)^6,7^. However, [^18^F]FDG PET has limitations in detecting HCC owing to variable FDG uptake^8,9^. Accordingly, immunoPET has shown potential for cancer management by assessing the expression of heterogeneous tumor antigens, such as GPC3, PSMA, HER2, PD-1 and PD-L1 (ref. ^6^). The mechanism of current immunoPET-based cancer management still confronts the fundamental challenge that antigen expression is particularly controversial in predicting responders^10^. Meanwhile, regarding ICI-based combination therapy, given the role of the VEGF pathway in maintaining an immunosuppressive tumor microenvironment and the existing evidence of anti-angiogenic efficacy of VEGF pathway inhibitors in HCC, it is reasonable to combine VEGF pathway inhibitors and ICIs in HCC^11^. Additionally, there are reported combination therapies using different ICIs, such as anti-CTLA-4 (ipilimumab) plus anti-PD-1 (nivolumab), anti-CTLA-4 (tremelimumab), and anti-PD-L1 (durvalumab)^12,13^. Summarizing the mechanisms of the aforementioned therapeutic methods raises two questions. First, although the current ICI-based combination therapies focus on multiple targets, how do we utilize PET imaging, which reflects a single feature, to evaluate therapeutic response? Second, recent ICI-based therapies activate “target A” triggering cancer cell death, whereas evaluating “target B” of dying cells by imaging is used to assess tumor response. Is there the same biomarker for reflecting and visualizing dying cells for achieving the direct valuation of ICI-based therapy response?

CD8^+^ T cells activated with ICIs have a central role in cancer immunotherapies, demonstrating the ability to eradicate cancer cells^14^. For example, after ICIs block the negative regulatory pathways that usually dampen T cell responses, T cells activated by antigen-presenting cells regain the ability to re-engage with tumor cells and release cytokines, ultimately leading to the death of tumor cells^15^. Recent work suggests that interferon-gamma (IFNγ) released from CD8^+^ T cells promotes cancer cell ferroptosis by suppressing the expression of SLC3A2 and SLC7A11, two subunits of the glutamate–cystine antiporter system x_C_^−^ (ref. ^16^). Ferroptosis regulates cell death by inducing iron-dependent lipid peroxide accumulation inside the cell^17^. Previous research suggested that DNA damage and repair are not involved in the ferroptosis-induced anticancer effect^18^. The sophisticated capability of DNA damage repair is regarded as a key hallmark in HCC, driving oncogenesis, progression, and therapeutic resistance^19^. Thus, ICI-triggered ferroptosis provides a potential therapeutic target for improving HCC management. Meanwhile, Olaparib, a poly (ADP-ribose) polymerase inhibitor (PARPi), inhibits PARP-mediated alternative end-joining repair, thereby converting single-strand breaks into lethal double-strand breaks for treating cancers with *BRCA1* and *BRCA2* mutations^20^. An increasing number of studies have revealed that Olaparib exhibits potential in collaborative treatment in BRCA-proficient HCC. For example, Olaparib sensitizes the BRCA-proficient HCC to irradiation, DDX11-targeted therapy, Sorafenib resistance case, and anti-androgen enzalutamide^21–24^. Interestingly, the updated research revealed that Olaparib promotes ferroptosis via repressing SLC7A11 (ref. ^18^).

Recently, we reported that ^18^F-5-fluoro-aminosuberic acid (denoted as [^18^F]FASu) PET imaging showed potential for evaluating SLC7A11 expression during HCC formation^8^. Compared with [^18^F]FDG PET, [^18^F]FASu PET imaging demonstrated higher sensitivity for HCC diagnosis and enabled the early characterization of pathological changes during HCC formation. Herein, we hypothesize that the combination of Olaparib and ICIs synergistically promotes ferroptosis in HCC, endowing SLC7A11 as the same target for combination therapy and evaluation of therapeutic response by [^18^F]FASu PET imaging. There are two important aspects reported in this PET image-guided combination therapy strategy. First, the combination of ICIs and Olaparib was utilized to promote ferroptosis for HCC treatment in a synergistic manner, thereby endowing the ferroptosis biomarker for subsequent imaging evaluations of therapeutic response. Second, the rational integration of therapy-induced ferroptosis with the imaging target enables direct evaluation of therapeutic response.

## Materials and methods

### [^18^F]FASu synthesis and characterization

[^18^F]FASu was synthesized via an optimized procedure adapted from prior literature. Specific details are provided in the Supplementary information. The final product was determined by radio-high-performance liquid chromatography (Supplementary Fig. 1). Decay-corrected radiochemical yield (RCY) was 18 ± 6% (*n* = 5), radiochemical purity was greater than 99%, and molar activity was 24 ± 4 GBq/mmol (*n* = 5).

### Colony formation assay

Hepa1-6 cells were seeded at a density of 800 per well in 6-well plates. After incubation for 24 h, they were treated with dimethyl sulfoxide, Olaparib (CAS: 763113-22-0, 5 μM), IFNγ (100 ng/ml), or a combination of Olaparib and IFNγ at the set concentrations for 48 h. Then, the medium was removed, fresh medium was added, and the cells were cultured for another 9 days. After the culture was completed, the cells were stained with 0.1% crystal violet methanol solution, and the colony formation was documented using a camera.

### Immunohistochemical assay

Paraffin sections were dewaxed through graded ethanol and rehydrated. Antigen retrieval was performed by high-pressure heating in ethylenediaminetetraacetic acid buffer (pH 9.0) for 90 s, followed by natural cooling to room temperature. Endogenous peroxidase activity was blocked with 3% H_2_O_2_ (30 min at room temperature). After tissue demarcation, sections were incubated with 10% normal goat serum (37 °C, 30 min) and subsequently with primary antibodies: Ki-67 (CST, 9129, 1:800), CD3 (Abcam, ab237721, 1:1,000), CD8 (Abcam, ab209775, 1:1,000), IFNγ (Affinity, DF6045, 1:400), SLC7A11 (Novus, NB300-318, 1:300), γ-H2AX (CST, 9718, 1:200; Abclonal, AP0687, 1:1,200), or 8-OHdG (Biotechne, 4354-MC-05080018-RP02, 1:2,000) at 4 °C overnight. Sections were then incubated with species-matched horseradish peroxidase-conjugated secondary antibodies (goat anti-rabbit or goat anti-mouse, 1:2,000; Abcam ab205718/ab205719) at 37 °C for 45 min. Signal development utilized DAB (3,3'-diaminobenzidine) with real-time monitoring counterstained with Harris hematoxylin. Sections were dehydrated through ethanol series, cleared with xylene substitute, and mounted with eco-friendly mounting medium for microscopic analysis using Olympus CX31 or digital scanning on Hamamatsu NanoZoomer S360.

### ROS detection

Hepa1-6 cells were stained with 2’,7’-dichlorodihydrofluorescein diacetate (H2DCFDA, E004-1-1, Nanjing Jiancheng, China) to assess reactive oxygen species (ROS) production. After treatment of each group of cells, H2DCFDA was added and incubated at 37 °C for 30 min. Five minutes before the incubation ended, Hoechst staining solution (C1027, Beyotime, China) was added to label the cell nuclei. Subsequently, the residual H2DCFDA and Hoechst staining solution were washed away with phosphate-buffered saline, and the cell fluorescence signals were observed under a fluorescence microscope (Olympus, Tokyo, Japan).

### Therapeutic and imaging protocols for animal tumor models

Male C57BL/6J mice aged 6–8 weeks were subcutaneously inoculated with 5 × 10^6^ Hepa1-6 cells in the subscapular region. On the 10th day after inoculation, 100 μg anti-PD-L1 antibody (10F.9G2, 100 μg dissolved in 100 μl phosphate-buffered saline, Bio X Cell) was intraperitoneally injected every 3 days along with 100 mg/kg Olaparib. Tumor volume was measured daily using a digital caliper and calculated according to the formula 1/2 × length × width^2^. Body weight was also recorded. [^18^F]FASu and [^18^F]FDG PET/CT imaging were performed on the 18th day and 20th day after tumor inoculation, respectively.

Male BALB/c nude mice aged 6–8 weeks were also subcutaneously inoculated with 5 × 10^6^ Hepa1-6 cells in the subscapular region. On the 10th day post-inoculation, 100 μg anti-PD-L1 antibody + 100 mg/kg Olaparib was intraperitoneally injected every 3 days. Tumor volume was measured every 3 days and calculated according to the same formula, and body weight was recorded simultaneously. [^18^F]FASu and [^18^F]FDG PET/CT imaging were performed on the 18th day and 20th day after tumor inoculation, respectively.

Mice were intravenously injected with 3.7 MBq of [^18^F]FASu or 3.7 MBq [^18^F]FDG (in-house PET center, Tongji Hospital, Tongji Medical College, Huazhong University of Science and Technology, Wuhan, China), respectively. A 10-min acquisition was performed 1 h later on a PET/CT scanner (uBio-EXPLORER, United Imaging, China). Data were reconstructed to get more high-resolution images using an ordered subsets expectation maximization (OSEM) algorithm (4 OSEM iterations, resolution: 1.4 mmHD) with scatter, attenuation, and decay corrections applied.

### Western blot analysis

Cell and tissue lysates were prepared using RIPA buffer supplemented with protease inhibitors (Theorell, Cat. no. TALR-013), with homogenization for tissues (SCIENTZ-48 homogenizer) followed by centrifugation (12,000×*g*, 15 min, 4 °C). Protein concentrations were quantified via BCA Assay (Thermo SCIENTIFIC, Cat. no. 23227) or NanoOne-1 (cell samples). Proteins (50 μg per lane) were resolved on 10% SDS–PAGE gels and transferred to NC membranes using a semi-dry system (Bio-Rad Trans-Blot Turbo). Membranes were blocked with rapid sealing solution (Yamay Biomedical Technology, Cat. no. PS108P) for 1 h and incubated overnight at 4 °C with primary antibodies: anti-SLC7A11 (Abcam Cat. no. ab307601, 1:1,000), anti-GPX4 (Proteintech Cat. no. 67763-1-Ig, 1:1,000), and anti-β-actin (Servicebio Cat. no. GB15001-100, 1:3,000). Horseradish peroxidase-conjugated secondary antibodies (CST Cat. no. 7074P2 and 7076P2, 1:10,000) were applied for 1 h at room temperature. Protein bands were visualized using enhanced chemiluminescence. All semi-quantitative results were normalized to β-actin as the loading control, with relative expression levels across experimental groups analyzed using ImageJ software (Version 1.54 g). Representative cropped blots are shown in the main figures. The full, uncropped scans of all western blots are provided in the Supplementary file (Supplementary Fig. 2).

### Statistical analysis

In vitro studies were performed with at least three independent biological replicates. For animal experiments, C57BL/6J or BALB/c nude mice were randomly assigned to groups following tumor cell inoculation, and mice that did not form tumors were excluded from subsequent analysis. The researchers were aware of group allocations during the study. Individual data points are displayed for all results. Statistical comparisons were conducted in GraphPad Prism 8. Continuous variables between two groups were compared using two-tailed unpaired *t*-tests or the Mann–Whitney *U* test, as appropriate. Comparisons among multiple groups were performed with one-way analysis of variance, followed by Tukey’s or Bonferroni’s test for post hoc analysis. Associations between gene expression levels were evaluated via Pearson’s correlation coefficient.

## Results

### The combination of anti-PD-L1 with Olaparib synergistically potentiates antitumor efficacy

**Fig. 1:**
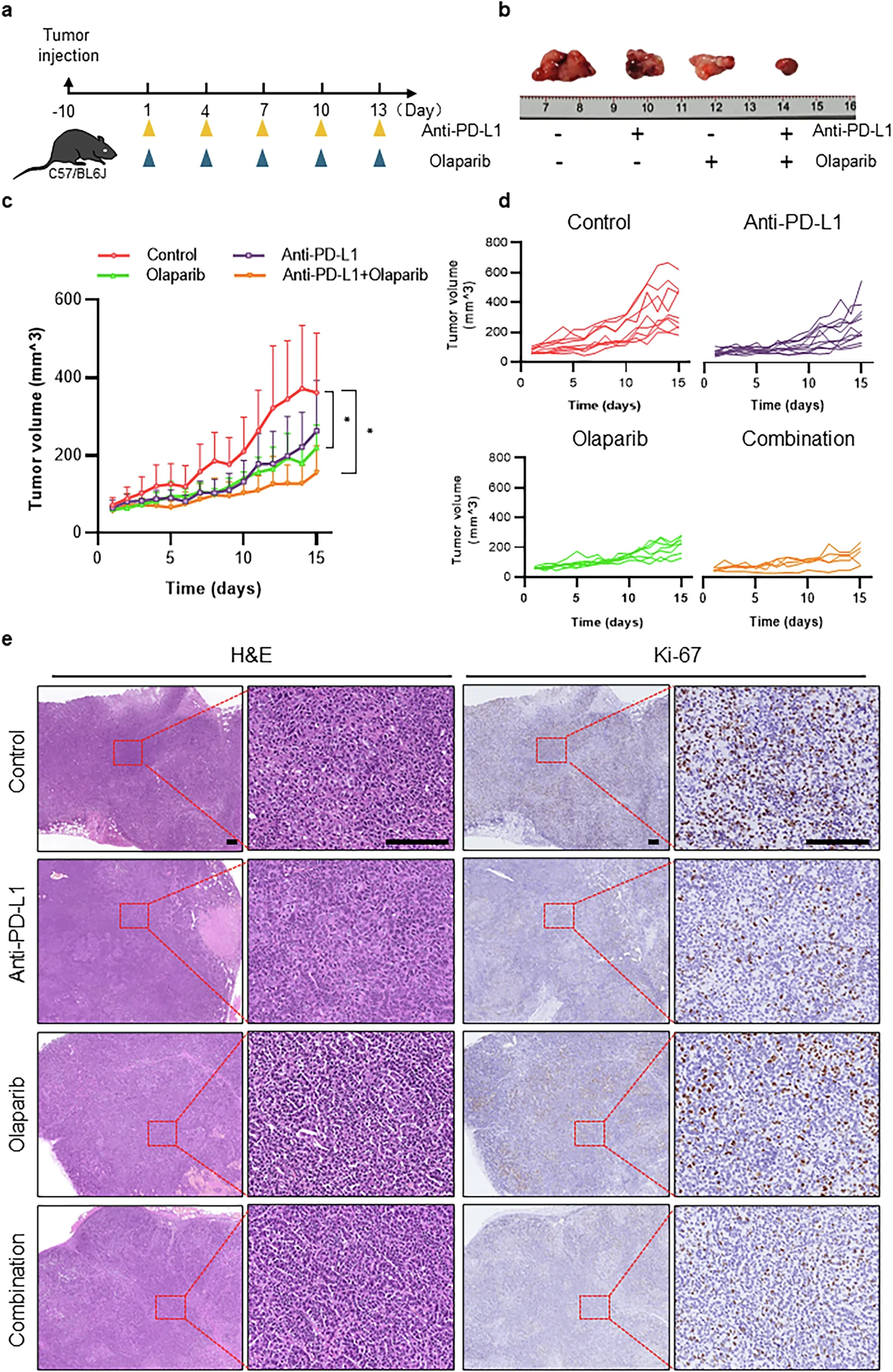
Combination of Olaparib and anti-PD-L1 therapy significantly enhances antitumor efficacy. **a** The treatment scheme of anti-PD-L1 antibody and Olaparib in Hepa1-6 tumor-bearing mice. **b** Representative photographs of tumor volumes on day 15. **c** Growth curves of tumors under different treatment groups (control group versus combination treatment group = 361.60 ± 153.30 mm^3^ versus 155.40 ± 69.51 mm^3^, *P* = 0.0157). **d** Individual tumor growth curves per treatment group (*n* ≥ 5 per group). **e** Immunohistochemical staining of hematoxylin and eosin (H&E) and Ki-67 on day 15. The expression of Ki-67 in the combination treatment group was significantly decreased compared with the control group. *P < 0.05. Scale bar, 200 μm.

For animal experiments, we used the Hepa1-6 tumor-bearing mouse model to carry out treatment and imaging according to the experimental protocol shown in Fig. 1a. At the end point, representative photos of tumor volume are presented in Fig. 1b. Compared with the control group, the tumor volume in the combined treatment group was significantly reduced (361.6 ± 153.3 mm^3^ versus 155.4 ± 69.5 mm^3^, *P* = 0.0157; Fig. 1c, d), indicating a favorable therapeutic effect.

Pathological analysis further confirmed the antitumor effect of the combined treatment. Immunohistochemical staining for Ki-67 on terminal tumor tissues demonstrated a marked reduction in Ki-67-positive cells in the combination treatment group (Fig. 1e). These results indicate that anti-PD-L1 combined with Olaparib demonstrated synergistic antitumor efficacy in the Hepa1-6 tumor-bearing mouse model.

**Fig. 2:**
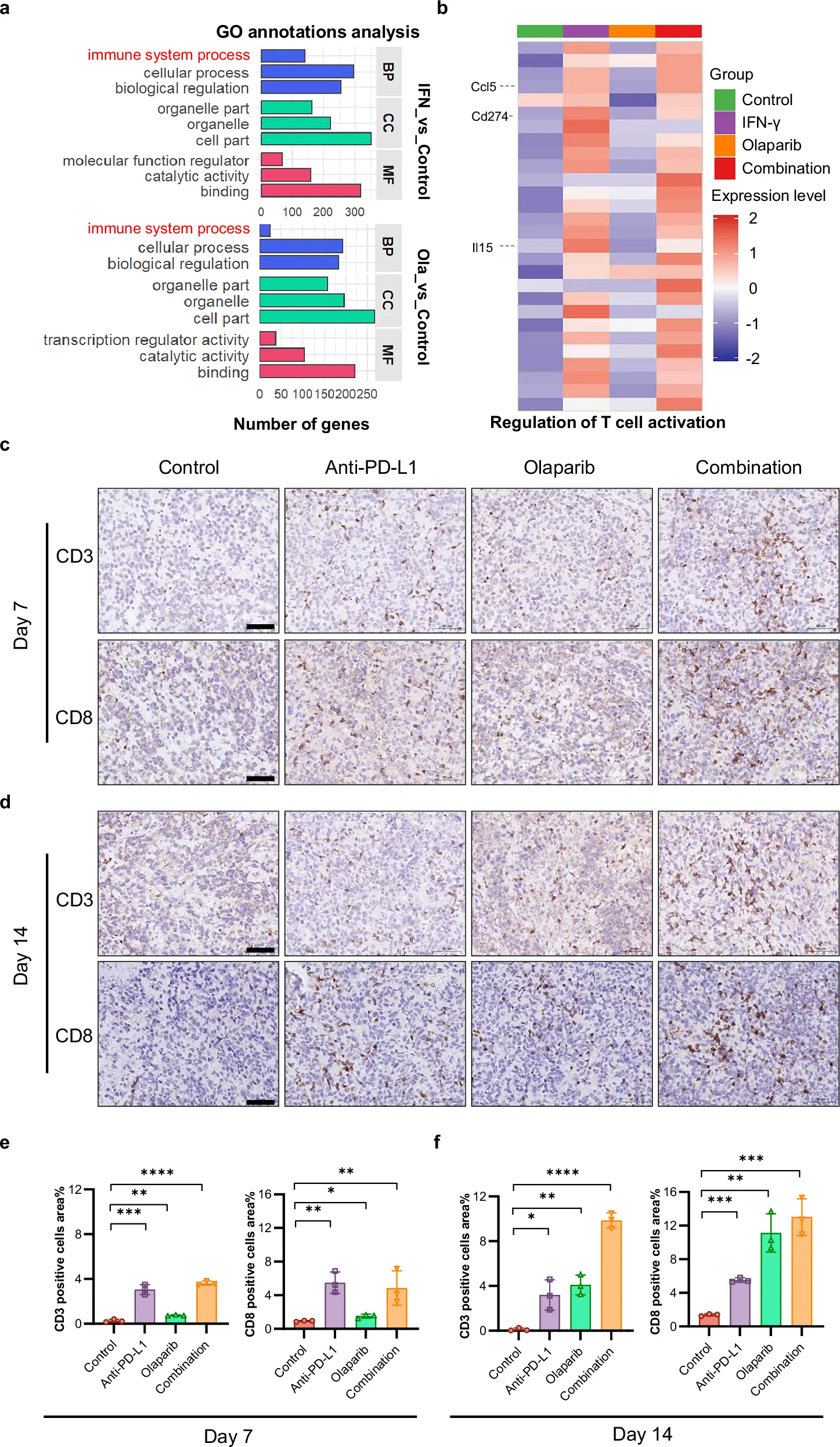
Olaparib combined with anti-PD-L1 therapy significantly increased CD3^+^ and CD8^+^ T cells infiltration. **a** Results of the Gene Ontology (GO) annotation analysis of the transcriptome. **b** Heatmap showing the expression levels of genes related to regulation of T cell activation in Hepa1-6 cells following different treatments. **c** Representative histological images of tumor sections stained with anti-CD3 and anti-CD8 antibodies on day 7. **d** Representative histological images of tumor sections stained with anti-CD3 and anti-CD8 antibodies on day 14. **e** Quantitative analysis of CD3 and CD8 expression levels (percentage of positive area) by immunohistochemistry on day 7. **f** Quantitative analysis of CD3 and CD8 expression level (percentage of positive area) by immunohistochemistry on day 14. Scale bar, 200 μm. BP, biological process; CC, cellular component; IFNγ, interferon-gamma; MF, molecular function.

Gene Ontology (GO) annotation analysis of the Hepa1-6 cell transcriptome revealed that the ‘immune system process’ term was enriched after treatment with IFNγ or Olaparib (Fig. 2a). Furthermore, heatmap visualization of the gene set (GO: Regulation of T cell activation) associated with the regulation of T cell activation revealed markedly elevated expression levels of relevant genes (including Ccl5, Cd274, and Il15) after distinct drug treatments (Fig. 2b). To further elucidate the underlying mechanisms, we analyzed immune infiltration in tumors at different time points. At day 7 post-treatment, both anti-PD-L1 and PARPi monotherapies enhanced antitumor immune responses, with synergistic effects observed in the combination group, as evidenced by increased CD3^+^ and CD8^+^ T cells (CD8-positive cells area%: control group = 0.93 ± 0.07, anti-PD-L1 group = 5.49 ± 1.28, Olaparib group = 1.52 ± 0.24, combination group = 4.86 ± 2.05) (Fig. 2c, d). At day 14, although anti-PD-L1 monotherapy showed stable levels of CD3 and CD8 positivity, PARPi monotherapy led to a further increase in these markers (CD8-positive cells area%: control group = 1.38 ± 0.13, anti-PD-L1 group = 5.52 ± 0.27, Olaparib group = 11.13 ± 2.28, combination group = 13.02 ± 2.19) (Fig. 2e, f). This suggests that PARPi monotherapy may continuously promote T cell recruitment or activation in the later stages of treatment.

### [^18^F]FASu PET/CT reflects the therapeutic efficacy of anti-PD-L1 combined with Olaparib

**Fig. 3:**
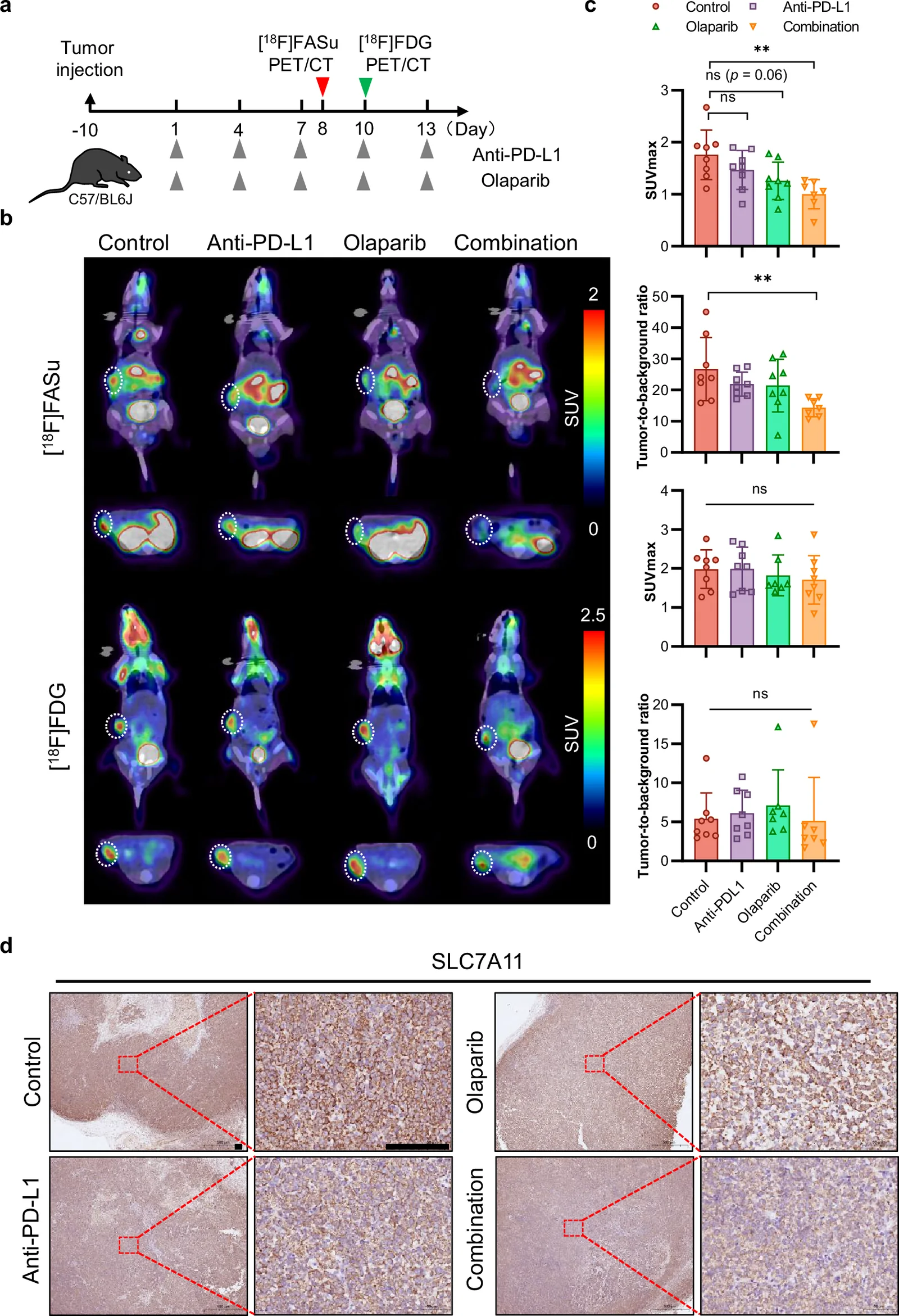
[^18^F]FASu PET/CT directly evaluates the therapeutic efficacy of anti-PD-L1 combined with Olaparib. **a** Schematic diagrams of imaging for Hepa1-6 tumor-bearing mice. **b** Uptake of [^18^F]FASu and [^18^F]FDG in Hepa1-6 tumor-bearing mice with different treatments at 1 h after injection. **c** Quantitative analysis of [^18^F]FASu and [^18^F]FDG with different treatments at 1 h after injection. The SUVmax in the combination treatment group was significantly decreased (control group versus combination treatment group = 1.76 ± 0.48 versus 1.00 ± 0.28, *P* = 0.0027). The tumor-to-background ratio was also reduced after treatment (control group versus combination treatment group = 26.71 ± 10.14 versus 14.31 ± 2.87, *P* = 0.0082). There was no statistically significant difference in either SUVmax or tumor-to-background ratio in [^18^F]FDG PET/CT (*P* > 0.05). **d** Representative histological images of tumors stained with anti-SLC7A11 antibody. The expression of SLC7A11 was significantly decreased in the combination treatment group. *P < 0.05, **P < 0.01, ***P < 0.001, ****P < 0.0001; ns, not significant. Scale bar, 200 μm.

The PET/CT imaging was performed at designated time points: [^18^F]FASu for oxidative stress assessment on day 8 post-treatment and [^18^F]FDG for metabolic activity evaluation on day 10 post-treatment (Fig. 3a). Visual assessment revealed distinct differences in tumor tracer uptake on PET/CT images after therapy (Fig. 3b). Compared with the control group, the SUVmax of the combined treatment group was significantly lower (control group versus combination group = 1.76 ± 0.48 versus 1.00 ± 0.28, *P* = 0.0027), and similarly, the tumor-targeted uptake ratio (TBR) was also significantly lower (control group versus combination group = 26.71 ± 10.14 versus 14.31 ± 2.87, *P* = 0.0082; Fig. 3c). However, there were no statistical differences in SUVmax and TBR between [^18^F]FDG PET/CT and the control group (*P* > 0.05; Fig. 3c). The tumor pathological staining at the intermediate time points revealed that the expression of SLC7A11 was significantly decreased in the combined treatment group (Fig. 3d). Furthermore, we found a correlation between SUVmax values and pathological CD8-positive area (*r* = −0.8095, *P* = 0.0218; Supplementary Fig. 3).

In the cellular uptake assay, cells treated with different drugs were incubated with [^18^F]FASu for 1 h. The combination treatment group exhibited lower counts per minute, which is consistent with the in vivo animal experimental results (Supplementary Fig. 4).

### T cell deficiency abrogates the sensitization effect of PARPi on anti-PD-L1 therapy

**Fig. 4:**
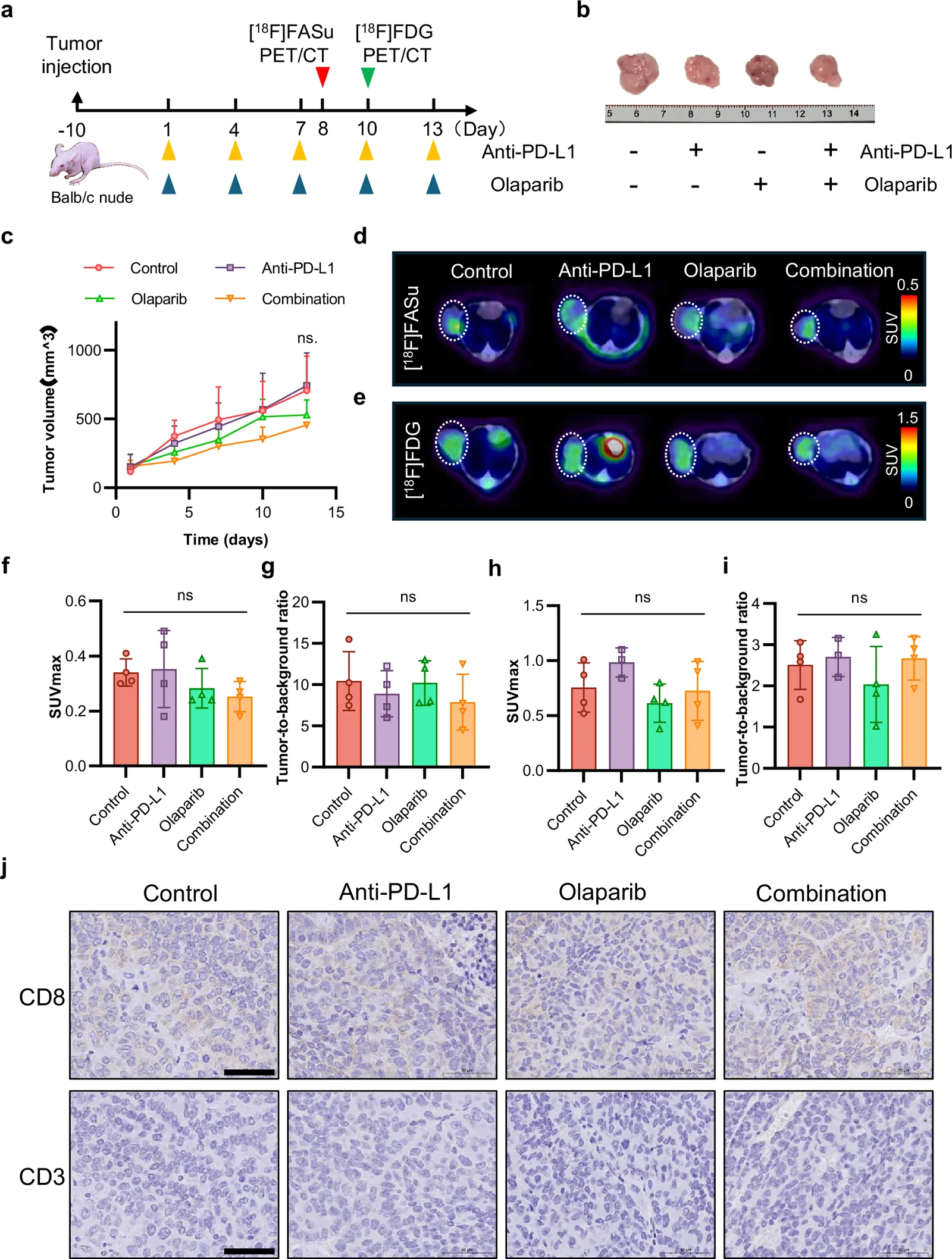
T cell deficiency abrogates the therapeutic efficacy of combination therapy of Olaparib and anti-PD-L1. **a** Schematic diagrams of the treatment and imaging regimen for BALB/c nude mice bearing Hepa1-6 tumors. **b** Representative photographs of tumor volumes at day 15. **c** Growth curves of tumors under different treatment groups. **d**, **e** Uptake of [^18^F]FASu and [^18^F]FDG at 1 h after injection. Quantitative analysis of [^18^F]FASu (parts **f** and **g**) and [^18^F]FDG (parts **h** and **i**) at 1 h after injection. There were no statistically significant differences in the SUVmax and tumor-to-background ratio values of [^18^F]FASu and [^18^F]FDG PET/CT among the groups (*P* > 0.05). **j** Representative histological images of CD3 and CD8 within different treatment tumors on day 15. ns, not significant. Scale bar, 50 μm.

CD8⁺ T cells had a key role in the treatment with Olaparib combined with anti-PD-L1. To further verify its dependence, we evaluated the efficacy of this combined treatment in an immune-deficient mouse model and analyzed the imaging and pathological results. The administration and imaging time points of this model were consistent with those of the C57BL/6J group (Fig. 4a).

The results showed that, compared with the control group, the tumor volume in the combined treatment group did not exhibit a significant reduction (Fig. 4b, c, *P* > 0.05). Additionally, the [^18^F]FASu and [^18^F]FDG PET/CT imaging analysis revealed no statistically significant differences in SUVmax and TBR values among the groups (Fig. 4d–i).

We also evaluated CD3^+^ and CD8^+^ T cells in immunodeficient models to underscore the critical role of T cells in the anti-PD-L1 and Olaparib combination therapy. Pathological results revealed no appreciable expression of CD3 and CD8 across all experimental groups (Fig. 4j).

### Anti-PD-L1-mediated IFNγ synergizes with Olaparib to trigger ferroptosis by escalating oxidative stress and impairing DNA damage repair

**Fig. 5:**
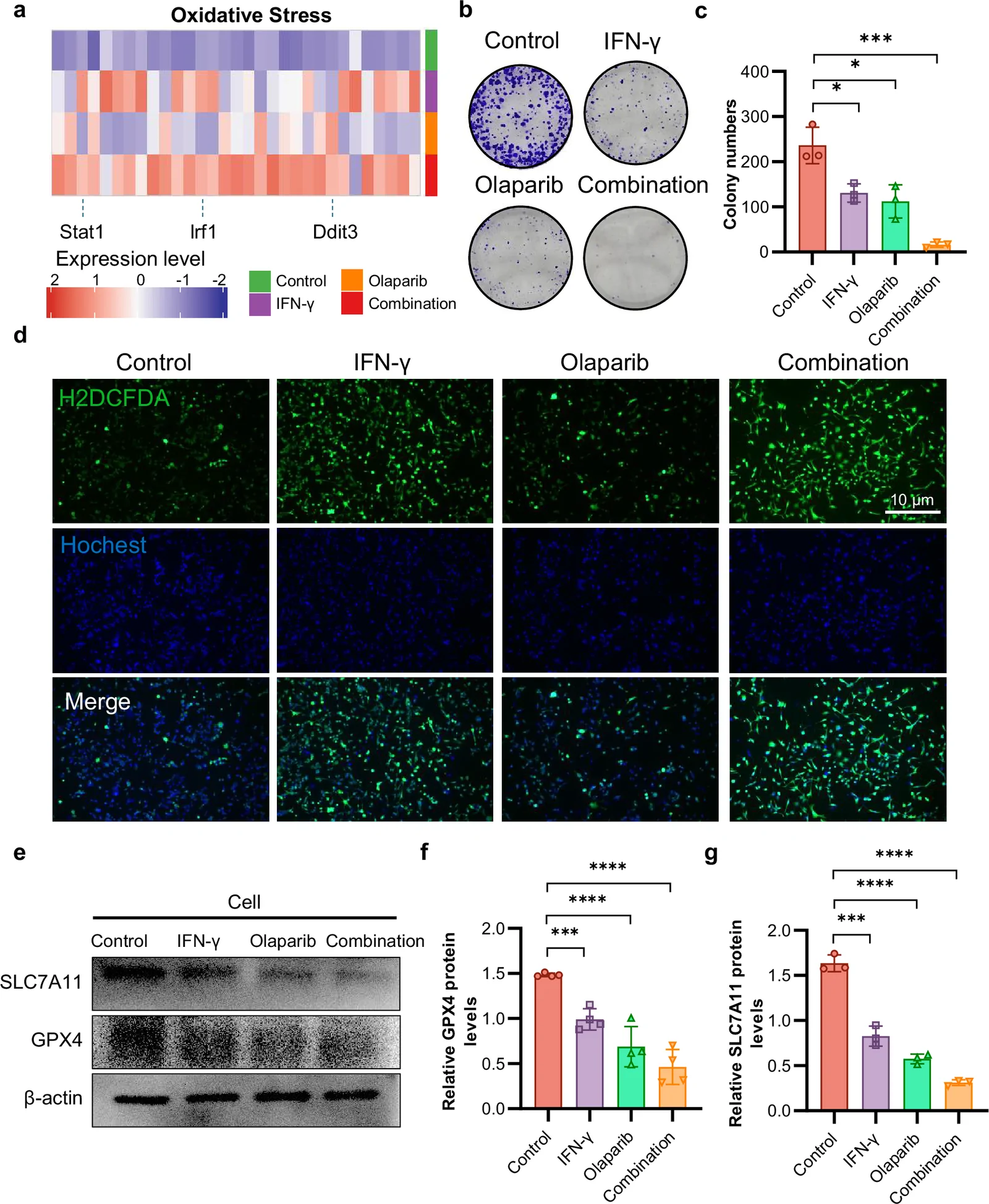
Olaparib and IFNγ synergistically induce ferroptosis through the suppression of SLC7A11 and GPX4. **a** Heatmap showing the expression levels of genes related to regulation of oxidative stress in Hepa1-6 cells following different treatments. **b** Representative images of clonogenic assay in Hepa1-6 cells treated with dimethyl sulfoxide (DMSO), Olaparib, interferon-gamma (IFNγ), and Olaparib in combination with IFNγ, respectively. **c** Quantification of clonogenic survival fractions in Hepa1-6 cells subjected to the indicated treatments. **d** Representative immunofluorescence images of reactive oxygen species in Hepa1-6 cells treated DMSO, Olaparib, IFNγ, and Olaparib in combination with IFNγ, respectively. **e** Western blot analysis of SLC7A11 and GPX4 protein expression (β-actin as loading control) in cultured Hepa1-6 cells. **f**, **g** Quantification of western blot analysis reveals that the combination group significantly downregulates SLC7A11 and GPX4 compared with the monotherapy groups, indicating synergistic induction of ferroptosis (*P* < 0.05). *P < 0.05, ***P < 0.001, ****P < 0.0001.

Heatmap of the Hepa1-6 transcriptome analysis under different treatments reveals elevated expression of oxidative stress-related genes, with particularly pronounced upregulation in the IFNγ and combination therapy groups (including Stat1, Irf1, and Ddit3) (Fig. 5a). To explore the potential mechanism of the antitumor effect of Olaparib combined with anti-PD-L1, we analyzed the influence of different treatments on cell colony formation ability. The results showed that compared with the control group, both Olaparib and IFNγ treatments significantly inhibited cell colony formation ability, and the inhibitory effect of the combined treatment group was more pronounced (Fig. 5b). Semi-quantitative results demonstrated synergistic killing of HCC cells by IFNγ and Olaparib (Fig. 5c).

Furthermore, we examined the levels of ROS under different treatment conditions. In the immunofluorescence experiment, the ROS level in Hepa1-6 cells treated with IFNγ was higher than that in the control group. More importantly, Olaparib alone could also induce a certain degree of ROS production, whereas in the combined treatment group, Olaparib and IFNγ together substantially enhanced ROS production (Fig. 5d). The above results suggest that Olaparib combined with IFNγ may mediate tumor cell killing through the ferroptosis mechanism. Western blot analysis demonstrated protein expression levels of SLC7A11 and GPX4 in cultured cells (β-actin as loading control) across four experimental groups: control, IFNγ monotherapy, Olaparib monotherapy, and combination therapy (Fig. 5e and Supplementary Fig. 2a). Quantitative analysis revealed significant downregulation of both SLC7A11 and GPX4 in the combination therapy group compared with monotherapy (*P* < 0.05), indicating synergistic induction of ferroptosis (Fig. 5f, g).

**Fig. 6:**
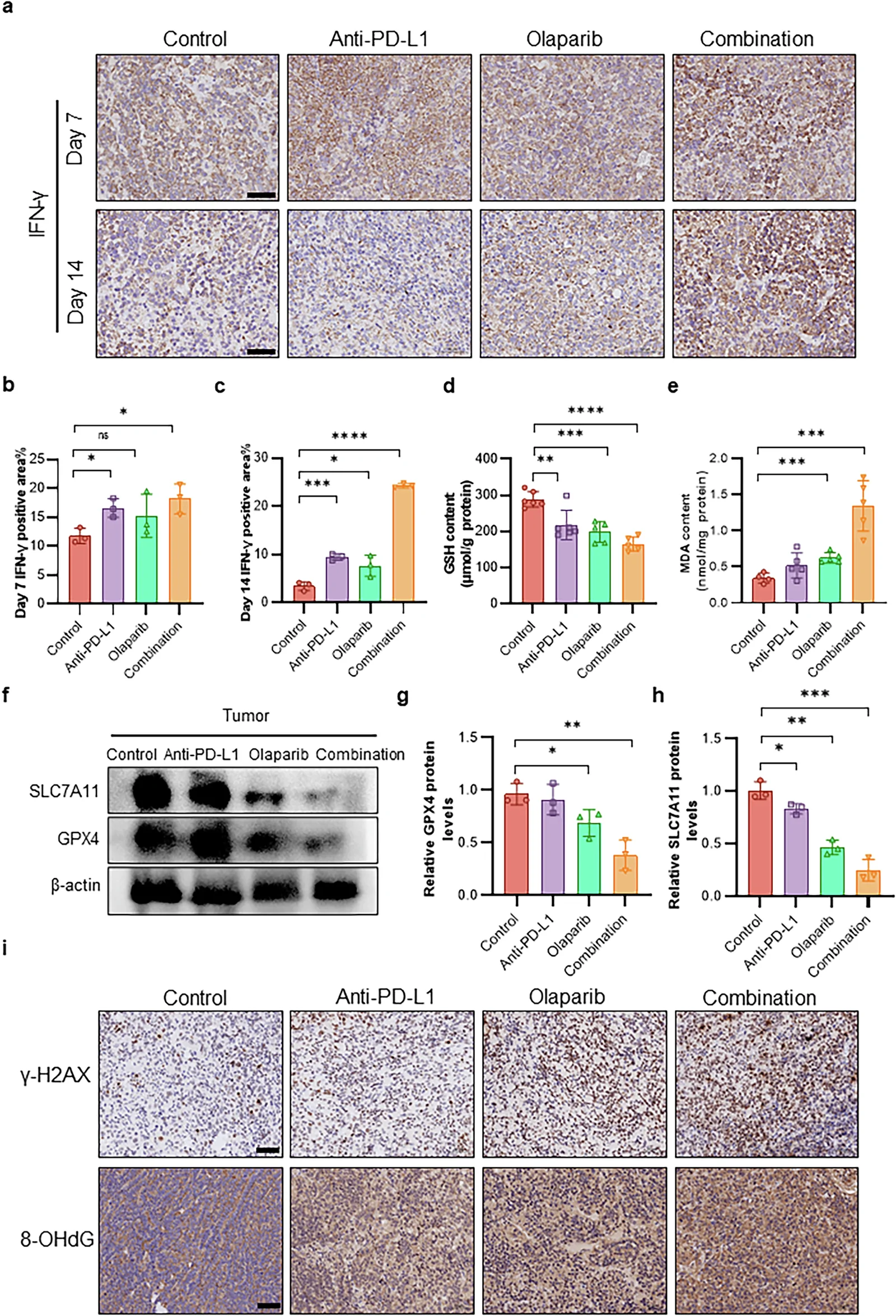
IFNγ elicited by Olaparib and anti-PD-L1 provokes oxidative stress and DNA damage. **a** Representative histological images of interferon-gamma (IFNγ) in different treatment tumors at day 7 and day 14. **b** Quantitative analysis revealed significantly higher IFNγ expression in the combination group compared with the control group (11.79 ± 1.32 versus 18.19 ± 2.60, *P* < 0.05). **c** Quantitative analysis of IFNγ expression at day 14 (control group versus combination treatment group = 3.47 ± 0.92 versus 24.30 ± 0.44, *P* < 0.05). **d**, **e** The content of glutathione (GSH) and malondialdehyde (MDA) in Hepa1-6 tumors. **f**, Western blot analysis of SLC7A11 and GPX4 (β-actin loading control). **g**, **h** Quantitative analysis of GPX4 and SLC7A11 protein levels. **i**, Representative histological images of γ-H2AX and 8-OHdG. *P < 0.05; **P < 0.01; ***P < 0.001; ****P < 0.0001. ns, not significant. Scale bar, 50 μm.

At various time points, we analyzed IFNγ expression levels via immunohistochemistry (IHC) in the C57BL/6J animal models. On day 7, both anti-PD-L1 and Olaparib monotherapies increased IFNγ infiltration within tumors to varying degrees, with the combination group exhibiting the highest level (Fig. 6a, b). By day 14, IFNγ expression had decreased in both monotherapy groups and the control groups yet remained elevated in the combination group (Fig. 6c). This indicates a synergistic regulation of IFNγ by the combination therapy, in which anti-PD-L1 promotes its production and Olaparib may potentiate its signaling, together leading to sustained immune activation within the tumor.

Concurrently, we assessed changes in ferroptosis-related markers (GSH and MDA). Inhibition of system x_C_^−^ reduced the level of cysteine, the rate-limiting precursor for GSH synthesis. Consequently, GSH content decreased in both monotherapy groups and was most significantly reduced in the combination group (Fig. 6d). Similarly, MDA levels were elevated in monotherapy groups and reached the highest level in the combination group, indicating that enhanced ferroptosis serves as a potential mechanism mediating synergistic antitumor effects (Fig. 6e).

We further validated decreased expression of GPX4 and SLC7A11 in tumors. Both proteins showed markedly lower expression in the combination group compared with controls (Fig. 6f, h and Supplementary Fig. 2b). Finally, the DNA damage marker γ-H2AX and the oxidative damage marker 8-OHdG were more highly expressed in the treated groups. By contrast, control groups demonstrated fewer DNA double-strand breaks and less oxidative stress (Fig. 6i).

## Discussion

In this work, we investigated a novel therapeutic approach combining anti-PD-L1 with Olaparib for the treatment of HCC. Notably, prominent synergistic effects were observed in immunocompetent C57BL/6J mouse models, whereas no significant therapeutic efficacy was detected in immunodeficient animal models. Pathological analysis further revealed that the PARPi Olaparib induced sustained infiltration of CD3^+^ and CD8^+^ T cells, suggesting that partially T cell-dependent antitumor immunity contributes to the efficacy of the combination therapy. Both anti-PD-L1 and Olaparib exerted tumor-killing effects by suppressing SLC7A11 expression, thereby triggering ferroptosis. In vitro experiments demonstrated that the combination therapy substantially increased intracellular ROS levels while downregulating the expression of SLC7A11 and GPX4 proteins. Consistent with these findings, in vivo studies showed increased IFNγ and MDA content, alongside decreased GSH levels, in tumors treated with the combination therapy. To non-invasively evaluate treatment response, we utilized [^18^F]FASu, a PET tracer targeting SLC7A11. The development of [^18^F]FASu, along with its key properties of specificity and non-interference with excitatory amino acid transporter (EAAT), has been well established in previous research^25^. Herein, we confirmed the insensitivity of tracer to EAAT inhibition and applied this tracer to monitor therapeutic efficacy (Supplementary Fig. 5a). Significantly reduced maximum standard uptake values (SUVmax) in the combination group suggest that [^18^F]FASu PET is a promising strategy for assessing the efficacy of anti-PD-L1 plus Olaparib combination therapy. The correlation among SLC7A11 expressions determined via pathological analysis and SUVmax further strengthens the biological interpretation of [^18^F]FASu (*r* = 0.8909, *P* = 0.0011, *n* = 10; Supplementary Fig. 5b). These findings provide evidence for the potential of this novel therapeutic approach and highlight the value of [^18^F]FASu PET as a tool for monitoring treatment response.

### Combination of ICIs and Olaparib was used to promote ferroptosis for HCC treatment in a synergistic manner, paving the way for precise imaging evaluations

T cells utilize the co-inhibitory receptors of PD-1 and CTLA-4 to balance immunity against foreign antigens and self-tolerance. In the circumstance of cancer, cancer cells upregulate related inhibitory molecules to induce tumor tolerance and T cell exhaustion^26^. Accordingly, ICIs, including CTLA-4, PD-1, and PD-L1 antibodies, can block immunosuppressive crosstalk between cancer cells and T cells after binding to the above co-inhibitory receptors, thereby reactivating the T cell immune response against cancer cells. However, monotherapy with ICIs is still hindered by limitations such as reduced efficacy and the emergence of resistance alongside potential systemic adverse effects. Thus, ICI-based combination therapy aims to achieve better outcomes by adding one more therapeutic agent. Typically, except for combination therapy with multiple ICIs, several other ingredients, such as tyrosine kinase inhibitors, anti-VEGF antibodies, and chemotherapeutic agents (for example, cisplatin, carboplatin, and paclitaxel), have been established to enhance the effect of ICI-based therapy^27,28^.

Recently, novel targets have been explored for ICI-based combination therapy. For example, MERTK inhibition enhanced the efferocytosis of dendritic cells and macrophages and modulated T cell activity by targeting adenosine signaling, the IDO1 pathway, and glucose and fatty-acid metabolism^29,30^. Undoubtedly, the discovery of those combinations involving multiple targets or biomarkers would revolutionize cancer treatment. Nevertheless, the employment of multiple biomarkers adds complexity to therapeutic response, thereby rendering precise imaging evaluations increasingly challenging.

Inspired by the novel mechanism that both ICIs and Olaparib can trigger ferroptosis, we hypothesize that the combination of ICIs and Olaparib synergistically promotes ferroptosis for HCC treatment by targeting a single target biomarker, SLC7A11, thereby benefiting subsequent imaging assessment. Building on evidence from the literature that PARPi can upregulate PD-L1 expression, thereby sensitizing tumors to PD-L1 blockade, we implemented this combined therapeutic approach in HCC models^31^. As shown in Fig. 1, the combination of PD-L1 antibody and Olaparib achieved a synergistic effect in eradicating tumors in Hepa1-6 tumor-bearing C57BL/6J  mice. By employing an immune deficiency model (Fig. 4), the Hepa1-6 tumor did not significantly shrink in the combination treatment group compared with the control group, indicating the synergistic effect of PD-L1 antibody and Olaparib in HCC treatment. To address interspecies differences, we evaluated the combination in human HepG2 cells. Treatment with IFNγ and Olaparib synergistically increased ROS levels (Supplementary Fig. 6a, b) and reduced cell viability (Supplementary Fig. 6c). In BALB/c nude mice, in which IFNγ was administered instead of anti-PD-L1 (Supplementary Fig. 6d), only the combination therapy significantly inhibited tumor growth (Supplementary Fig. 6e). The lack of efficacy with Olaparib monotherapy highlights a potential difference in ferroptosis susceptibility between cell types.

SLC7A11, the functional subunit of the cystine–glutamate antiporter system x_C_^−^ imports extracellular cystine for GSH synthesis. Its inhibition depletes GSH and cripples cellular antioxidant capacity, priming cells for ferroptosis^32^. GPX4, a GSH-dependent selenoenzyme, directly neutralizes toxic phospholipid hydroperoxides to halt lipid peroxidation. Loss of GPX4 activity permits lethal peroxidation to accumulate, executing ferroptosis cell death. The analysis of key markers further revealed the downregulation of SLC7A11 and GPX4 in the combination group (Figs. 5 and  6). The quantification of GSH and MDA contents, which are components involved in ferroptosis, further demonstrated the ferroptosis-induced anticancer effect (Fig. 6). In vitro ROS levels (Figs. 5 and  6) and in vivo DNA damage further confirmed the synergistic anticancer effect of the combination. Additionally, we performed rescue experiments targeting ferroptosis using the specific inhibitor Ferrostatin-1 (Supplementary Fig. 7). The results showed that Ferrostatin-1 reduced the ROS levels induced by the combination of Olaparib and IFNγ and reversed the therapeutic efficacy of the regimen, which is consistent with a ferroptosis-dependent mechanism.

The activity and infiltration of CD8-positive T cells in tumors are critical for ICI-based treatment. Notably, prior investigations have found that PARPi can promote lymphocytic infiltration within tumor tissues and counteract the immunosuppressive properties of the tumor microenvironment, which is associated with improved overall survival outcomes^33^. In Fig. 2, IHC staining revealed high upregulation of CD8 on day 7 and decreased upregulation of CD8 on day 14, following PD-L1 antibody treatment alone. By contrast, Olaparib alone showed a consistent increase in the upregulation of CD8. Previous work has reported that PARPi enhances lymphocyte infiltration in tumors and modulates the immunosuppressive tumor microenvironment^33^. The analysis of IHC staining demonstrated that the prolonged upregulation of CD8 was achieved by integrating Olaparib in the combination treatment. Therefore, our findings may provide a novel approach to improve outcomes of ICIs in HCC treatment.

Taken together, the combination of ICIs and Olaparib was utilized to promote ferroptosis for HCC treatment in a synergistic manner, thereby endowing the ferroptosis biomarker for subsequent imaging evaluations of therapeutic response.

### Rational integration of therapy-induced ferroptosis and imaging target enables the direct evaluation of therapeutic response

Evaluation of tumor response during ICI-based therapy is critical for HCC management. The features, such as changes in biological processes and the expression of biomarkers, are intrinsic mechanisms of tumor response post treatment^34–37^. However, the broad application of CT and MRI is commonly used to assess lesion morphology, which occurs in the delayed response^38^. Thus, a gap exists between the parameters of CT-based or MRI-based imaging assessments and the features of tumor response, impeding precise diagnosis and timely management of treatment plans.

PET imaging offers an alternative approach by detecting various features of cancer cells, including metabolic processes and biomarker expression. For example, besides [^18^F]FDG PET, the lipophilic demand in HCC allows PET imaging with [^11^C]-acetate, [^18^F]-fluorocholine, and [^11^C]-choline to visualize the lesions^8^. In addition, ImmunoPET could provide valuable information on tumors, including tumor antigens, immune cells, and inflammatory processes^6^. However, upon scrutinizing the ICI-based therapeutic modalities (Supplementary Tables 1 and 2), the features or targets for PET imaging are not directly related to therapy-induced ferroptosis. In detail, ICI-based therapy activates “target A” triggering cancer cell death, then visualizing the “target B” of dying cells by PET imaging. Obviously, this is a gap between PET imaging assessment and tumor response, as evaluated by CT or MRI. Herein, we explored the rational integration of therapy-induced ferroptosis and an imaging target by targeting SLC7A11, enabling the direct evaluation of therapeutic response by PET imaging. By comparing the SUVmax of PET imaging in an immune response-involved animal model (Fig. 3) and an immune deficiency model (Fig. 4), our results further confirm the value of SLC7A11 targeting PET imaging for evaluating therapeutic response. [^18^F]FASu exhibits greater specificity for system x_C_⁻ than the classical tracer [^18^F]FSPG, avoiding interference from other transporters (for example, EAATs)^25^. Superior alignment between therapeutic and imaging targets was compared with traditional PD-1 and PD-L1 tracers, enabling more direct monitoring of immunotherapy efficacy.

### Limitations and future perspectives

This study has several limitations, highlighting areas for future investigation. First, our study did not assess the acute and chronic toxicity associated with this novel therapeutic paradigm. Although the toxicities and side effects of anti-PD-L1 and Olaparib have been well characterized individually, further studies on the toxicity profile of their combination are essential to advance its translation into clinical practice. Second, regarding the mechanism of ferroptosis induced by Olaparib and anti-PD-L1, although we demonstrated the expression of related markers and confirmed distinct therapeutic responses in immune-deficient and normal mouse models, additional studies using ferroptosis inhibitors or transgenic animal models would further strengthen our findings. Third, the observed decrease in [^18^F]FASu accumulation in lesions following combination therapy suggests that establishing a baseline for [^18^F]FASu PET imaging before initiating combination therapy may be necessary for accurate evaluation of treatment response. This may also introduce an additional clinical burden.

[^18^F]FASu is currently at the preclinical stage of development. Although our data demonstrate its promising imaging properties and biological relevance in animal models, comprehensive toxicity, safety and human dosimetry studies that are essential prerequisites for clinical application have not yet been conducted. Although the biodistribution of [^18^F]FASu in animal models has been studied, we have supplemented the preclinical data with results obtained from C57BL/6J   normal mice (Supplementary Fig. 8 and Supplementary Table 3). The primary aim of this study was to establish the foundational mechanistic rationale and imaging efficacy of [^18^F]FASu for detecting oxidative stress in the tumor microenvironment. Its eventual translation to human use will require extensive future investigations, including first-in-human trials that serve to formally establish its safety profile and pharmacokinetics.

The proposed T cell–IFNγ–SLC7A11-ferroptosis axis is presented as a working model consistent with our data, but it has not been directly validated through functional experiments such as T cell depletion or IFNγ neutralization. Therefore, the causal role of T cells and IFNγ in this signaling cascade remains to be confirmed in future studies. Additional investigations using genetic knockout models or specific inhibitors would help further clarify the underlying mechanisms.

Nonetheless, the combination of anti-PD-L1 with Olaparib in BRCA wild-type HCC suggests a mechanism involving suppression of SLC7A11 and the subsequent promotion of ferroptosis, supporting the potential for therapeutic response monitoring via ferroptosis biomarker imaging. The SLC7A11-targeted PET tracer [^18^F]FASu provides a clinically translatable biomarker for monitoring treatment efficacy. These findings establish a novel theragnostic paradigm that integrates therapeutic and imaging pathways, allowing for direct evaluation of treatment response using PET imaging.

### Supporting information

All relevant experimental details, imaging datasets, and analysis scripts are included in the manuscript and in its Supporting information.

## Supplementary information

Supplementary Information

## Data Availability

The transcriptomics data are available in the Gene Expression Omnibus (GEO). RNA-seq data are available at the GEO under the accession number GSE329164.

## References

[1] Sung, H. et al. Global Cancer Statistics 2020: GLOBOCAN estimates of incidence and mortality worldwide for 36 cancers in 185 countries. *CA Cancer J Clin* **71**, 209–249 (2021). 10.3322/caac.21660 33538338

[2] Ruf, B., Heinrich, B. & Greten, T. F. Immunobiology and immunotherapy of HCC: spotlight on innate and innate-like immune cells. *Cell Mol Immunol* **18**, 112–127 (2021). 10.1038/s41423-020-00572-w PMC7852696 33235387

[3] Sangro, B. et al. Diagnosis and management of toxicities of immune checkpoint inhibitors in hepatocellular carcinoma. *J Hepatol* **72**, 320–341 (2020). 10.1016/j.jhep.2019.10.021 PMC7779342 31954495

[4] Dercle, L. et al. Emerging and evolving concepts in cancer immunotherapy imaging. *Radiology* **306**, 32–46 (2023). 10.1148/radiol.210518 36472538

[5] Hughes, D. J. et al. (18)F FDG PET/CT and novel molecular imaging for directing immunotherapy in cancer. *Radiology* **304**, 246–264 (2022). 10.1148/radiol.212481 35762888

[6] Wei, W. et al. ImmunoPET: concept, design, and applications. *Chem Rev* **120**, 3787–3851 (2020). 10.1021/acs.chemrev.9b00738 PMC7265988 32202104

[7] Cavo, M. et al. Role of (18)F-FDG PET/CT in the diagnosis and management of multiple myeloma and other plasma cell disorders: a consensus statement by the International Myeloma Working Group. *Lancet Oncol* **18**, e206–e217 (2017). 10.1016/S1470-2045(17)30189-4 28368259

[8] Xiong, F. et al. ^18^F-5-fluoro-aminosuberic acid PET/CT imaging of oxidative-stress features during the formation of DEN-induced rat hepatocellular carcinoma. *Theranostics* **15**, 141–154 (2025). 10.7150/thno.100467 PMC11667236 39744233

[9] Haug, A. R. Imaging of primary liver tumors with positron-emission tomography. *Q J Nucl Med Mol Imaging* **61**, 292–300 (2017). 10.23736/S1824-4785.17.02994-6 28686007

[10] Grossman, J. E., Vasudevan, D., Joyce, C. E. & Hildago, M. Is PD-L1 a consistent biomarker for anti-PD-1 therapy? The model of balstilimab in a virally-driven tumor. *Oncogene* **40**, 1393–1395 (2021). 10.1038/s41388-020-01611-6 PMC7906900 33500548

[11] Finn, R. S. et al. Atezolizumab plus bevacizumab in unresectable hepatocellular carcinoma. *N Engl J Med* **382**, 1894–1905 (2020). 10.1056/NEJMoa1915745 32402160

[12] Abou-Alfa, G. K. et al. Tremelimumab plus durvalumab in unresectable hepatocellular carcinoma. *NEJM Evid* **1**, EVIDoa2100070 (2022). 10.1056/EVIDoa2100070 38319892

[13] Yau, T. et al. Efficacy and safety of nivolumab plus ipilimumab in patients with advanced hepatocellular carcinoma previously treated with sorafenib: the CheckMate 040 Randomized Clinical Trial. *JAMA Oncol* **6**, e204564 (2020). 10.1001/jamaoncol.2020.4564 PMC7530824 33001135

[14] Ito, Y. et al. Addressing tumor heterogeneity by sensitizing resistant cancer cells to T cell-secreted cytokines. *Cancer Discov* **13**, 1186–1209 (2023). 10.1158/2159-8290.CD-22-1125 PMC10164097 36811466

[15] Wawrzyniak, P. & Hartman, M. L. Dual role of interferon-gamma in the response of melanoma patients to immunotherapy with immune checkpoint inhibitors. *Mol Cancer* **24**, 89 (2025). 10.1186/s12943-025-02294-x PMC11924818 40108693

[16] Wang, W. et al. CD8^+^ T cells regulate tumour ferroptosis during cancer immunotherapy. *Nature* **569**, 270–274 (2019). 10.1038/s41586-019-1170-y PMC6533917 31043744

[17] Yang, W. S. & Stockwell, B. R. Ferroptosis: death by lipid peroxidation. *Trends Cell Biol* **26**, 165–176 (2016). 10.1016/j.tcb.2015.10.014 PMC4764384 26653790

[18] Hong, T. et al. PARP inhibition promotes ferroptosis via repressing SLC7A11 and synergizes with ferroptosis inducers in BRCA-proficient ovarian cancer. *Redox Biol* **42**, 101928 (2021). 10.1016/j.redox.2021.101928 PMC8113041 33722571

[19] Hong, W. et al. Deciphering the immune modulation through deep transcriptomic profiling and therapeutic implications of DNA damage repair pattern in hepatocellular carcinoma. *Cancer Lett* **582**, 216594 (2024). 10.1016/j.canlet.2023.216594 38135208

[20] Chopra, N. et al. Homologous recombination DNA repair deficiency and PARP inhibition activity in primary triple negative breast cancer. *Nat Commun* **11**, 2662 (2020). 10.1038/s41467-020-16142-7 PMC7260192 32471999

[21] Chen, G., Zheng, D., Zhou, Y., Du, S. & Zeng, Z. Olaparib enhances radiation-induced systemic anti-tumor effects via activating STING-chemokine signaling in hepatocellular carcinoma. *Cancer Lett* **582**, 216507 (2024). 10.1016/j.canlet.2023.216507 38048841

[22] Cao, K. et al. Targeting DDX11 promotes PARP inhibitor sensitivity in hepatocellular carcinoma by attenuating BRCA2-RAD51 mediated homologous recombination. *Oncogene* **43**, 35–46 (2024). 10.1038/s41388-023-02898-x 38007537

[23] Yang, X. D. et al. PARP inhibitor Olaparib overcomes Sorafenib resistance through reshaping the pluripotent transcriptome in hepatocellular carcinoma. *Mol Cancer* **20**, 20 (2021). 10.1186/s12943-021-01315-9 PMC7824946 33485358

[24] Zhao, J. et al. Olaparib and enzalutamide synergistically suppress HCC progression via the AR-mediated miR-146a-5p/BRCA1 signaling. *FASEB J* **34**, 5877–5891 (2020). 10.1096/fj.201903045RR PMC7243671 32134529

[25] Colovic, M. et al. Comparative evaluation of [(18)F]5-fluoroaminosuberic acid and (4S)-4-3-[(18)F]fluoropropyl)-L-Glutamate as system xC-targeting radiopharmaceuticals. *J Nucl Med* **64**, 1314–1321 (2023). 10.2967/jnumed.122.265254 37116917

[26] Sadeghi Rad, H. et al. Understanding the tumor microenvironment for effective immunotherapy. *Med Res Rev* **41**, 1474–1498 (2021). 10.1002/med.21765 PMC8247330 33277742

[27] Shen, K. Y., Zhu, Y., Xie, S. Z. & Qin, L. X. Immunosuppressive tumor microenvironment and immunotherapy of hepatocellular carcinoma: current status and prospectives. *J Hematol Oncol* **17**, 25 (2024). 10.1186/s13045-024-01549-2 PMC11057182 38679698

[28] Shiravand, Y. et al. Immune checkpoint inhibitors in cancer therapy. *Curr Oncol* **29**, 3044–3060 (2022). 10.3390/curroncol29050247 PMC9139602 35621637

[29] Kraehenbuehl, L., Weng, C. H., Eghbali, S., Wolchok, J. D. & Merghoub, T. Enhancing immunotherapy in cancer by targeting emerging immunomodulatory pathways. *Nat Rev Clin Oncol* **19**, 37–50 (2022). 10.1038/s41571-021-00552-7 34580473

[30] Llopiz, D. et al. MERTK inhibition improves therapeutic efficacy of immune checkpoint inhibitors in hepatocellular carcinoma. *Oncoimmunology* **14**, 2473165 (2025). 10.1080/2162402X.2025.2473165 PMC11881874 40029206

[31] Jiao, S. et al. PARP inhibitor upregulates PD-L1 expression and enhances cancer-associated immunosuppression. *Clin Cancer Res* **23**, 3711–3720 (2017). 10.1158/1078-0432.CCR-16-3215 PMC5511572 28167507

[32] Dixon, S. J. et al. Ferroptosis: an iron-dependent form of nonapoptotic cell death. *Cell* **149**, 1060–1072 (2012). 10.1016/j.cell.2012.03.042 PMC3367386 22632970

[33] Liu, J. et al. Mitigating T cell DNA damage during PARP inhibitor treatment enhances antitumor efficacy. *Sci Transl Med* **17**, eadr5861 (2025). 10.1126/scitranslmed.adr5861 40333991

[34] Bansal, A., Goyal, S., Goyal, A. & Jana, M. WHO classification of soft tissue tumours 2020: an update and simplified approach for radiologists. *Eur J Radiol* **143**, 109937 (2021). 10.1016/j.ejrad.2021.109937 34547634

[35] Kim, K., Mercer, J., John, V., Mathew, S. & Kochhar, R. Imaging features of anal carcinoma after chemoradiation. *Radiographics* **45**, e240119 (2025). 10.1148/rg.240119 40080437

[36] Lokre, O. et al. Quantitative evaluation of lesion response heterogeneity for superior prognostication of clinical outcome. *Eur J Nucl Med Mol Imaging* **51**, 3505–3517 (2024). 10.1007/s00259-024-06764-0 PMC11445285 38819668

[37] Ge, T. et al. Crosstalk between metabolic reprogramming and epigenetics in cancer: updates on mechanisms and therapeutic opportunities. *Cancer Commun*(*Lond*) **42**, 1049–1082 (2022). 10.1002/cac2.12374 PMC9648395 36266736

[38] Seymour, L. et al. iRECIST: guidelines for response criteria for use in trials testing immunotherapeutics. *Lancet Oncol* **18**, e143–e152 (2017). 10.1016/S1470-2045(17)30074-8 PMC5648544 28271869

