# Supplementary material for: PET imaging enables direct evaluation of ferroptosis in HCC synergistically induced by anti-PD-L1 and Olaparib
Source: Exp Mol Med. 2026 Oct 9;58(10):3534–45. doi: 10.1038/s12276-026-01860-7 (PMC13737193; doi:10.1038/s12276-026-01860-7)
Supplement: Supplementary file 1 [file 12276_2026_1860_MOESM1_ESM.pdf]

Supplementary Information

PET imaging enables direct evaluation of ferroptosis in HCC synergistically induced by anti-PD-L1 and Olaparib

Feng Xiong[1,2,†], Cheng Xu[3,4,†], Pei Wang[1,2,†], Ni Li[6,†], Yilin Yang[1,2], Buchuan Zhang[1,2], Kijung Kwak[5], Shuang Song[1], Pengtao You[3,4,*], Xiaohua Zhu[1], Dalong Ni[6,7,*], Bo Yu[1,2,*]

1. Department of Nuclear Medicine, Tongji Hospital, Tongji Medical College, Huazhong University of Science and Technology, Wuhan, China;

2. Department of Nuclear Medicine, Tongji Hospital, Tongji Medical College and State Key Laboratory for Diagnosis and Treatment of Severe Zoonotic Infectious Diseases, Huazhong University of Science and Technology, Wuhan, China;

3. Hubei University of Chinese Medicine, Wuhan 430030, Hubei Province, China;

4. Hubei Key Laboratory of Resources and Chemistry of Chinese Medicine, Hubei University of Chinese Medicine, Wuhan 430030, Hubei Province, China;

5. University of Maryland School of Medicine, 655 W Baltimore St S, Baltimore, MD 21201, USA.

6. Department of Orthopaedics, Shanghai Key Laboratory for Prevention and Treatment of Bone and Joint Diseases, Shanghai Institute of Traumatology and Orthopaedics, Ruijin Hospital, Shanghai Jiao Tong University School of Medicine, Shanghai 200025, China

7. Suzhou Institute of Biomedical Engineering and Technology, Chinese Academy of Science, Suzhou 215163, China

†Equally contributed to this work.

∗**Corresponding authors.**

E-mails: Prof. Bo Yu, PhD: yuboxf@hotmail.com, +8613367278998; Prof. Dalong Ni, PhD: ndl12353@rjh.com.cn; Prof. Pengtao You: tptyou@hbucm.com.

1. Materials and methods

1.1 [^{18}F]FASu Synthesis

Identification and quality control of [^{18}F]FASu were performed by high-performance liquid chromatography (HPLC). The compound was characterized using reverse-phase HPLC on a Waters C18 column (5 μm, 250 × 4.6 mm) with a flow rate of 1 mL/min. A gradient elution was applied, starting with 0% solvent B at 0 min and increasing to 15% solvent B at 20 min (solvent A: 0.1% trifluoroacetic acid in H_2O; solvent B: acetonitrile). The corresponding chromatograms are provided in **Supplementary Fig. 1**.

1.2 Cellular Uptake and EAAT Inhibition Experiments

Experiments were performed using Hepa1-6 cells cultured in 6-well plates. Cells were treated for 16 hours with IFN-γ (100 ng/mL), Olaparib (1 μM), their combination, or an equivalent volume of DMSO as a control. After being washed twice with PBS, the cells were incubated with 0.2 MBq [^{18}F]FASu for 1 hour. Subsequently, the cells were rapidly washed twice with ice-cold PBS and lysed with 1 M NaOH for 1 minute. The cell lysate was collected, and its radioactivity was measured using a γ-counter, with the results expressed as counts per minute (CPM) for further analysis.

For the EAAT inhibition assay, Hepa1-6 cells were co-incubated with 1 mM *L*-trans-pyrrolidine-2,4-dicarboxylic acid (PDC, Sigma-Aldrich, P7575) for 16 h. After a 60-minute tracer (0.2 MBq [^{18}F]FASu) incubation period, the assay buffer was removed to terminate uptake. The cells were then rapidly washed twice with ice-cold PBS and lysed with 1 M NaOH for 1 min. Finally, the cell lysate was collected for analysis.

1.3 Human Cell ROS Detection and Animal Model Experiments

To assess reactive oxygen species (ROS) production, HepG2 cells were loaded with 10 μM 2',7'-dichlorodihydrofluorescein diacetate (H2DCFDA; HY-D0940, MCE, USA) and incubated at 37°C for 30 min. Five minutes prior to the end of this incubation, Hoechst staining solution (C0030, Solarbio, China) was added to label cell nuclei.

Following incubation, cells were washed with phosphate-buffered saline (PBS) to remove residual probes, and fluorescence signals were visualized using a fluorescence microscope.

To establish tumors, male BALB/c Nude mice (aged 6–8 weeks) were subcutaneously inoculated in the subscapular region with 1×10^7 HepG2 cells. Beginning on day 7 post-inoculation, mice received intraperitoneal injections of 1.25×10^5 U of recombinant human IFN-γ (300-02, PeproTech) in 100 μL PBS every three days, co-administered with 100 mg/kg Olaparib. Tumor volume and body weight were recorded every three days, with volume calculated using the formula: $1/2 \times \text{length} \times \text{width}^2$.

1.4 Ferroptosis Inhibition Assay

The Hepa1-6 cells were first pretreated with 2 μM Ferrostatin-1 (Fer-1, HY-100579, MCE, USA) for 14 h, followed by co-incubation with various drugs for 24 h. After treatment, the cells were washed thrice with ice-cold PBS and subjected to H2DCFDA and Hoechst staining.

1.5 Cell Proliferation and Viability Assay.

Cell viability in all experiments was assessed using a CCK-8 kit (BS350B, Biosharp). Following the respective drug treatments, 10 μL of CCK-8 solution was added to each well. The plates were then returned to the cell culture incubator for an additional 30 minutes. Finally, the absorbance at 450 nm was measured using a microplate reader.

1.6 Biodistribution Studies

Hepa1-6 tumor-bearing mice received an intravenous injection of 3.6 MBq [^{18}F]FASu. Following a 1-hour free distribution period, the animals were euthanized via CO_2 asphyxiation. Blood samples were immediately collected by cardiac puncture. Subsequently, target organs and tissues were dissected, rinsed with phosphate-buffered saline (PBS), gently blotted dry, and weighed; their radioactivity was then measured,

normalized to the injected dose, and expressed as percentage injected dose per gram of tissue (%ID/g).

1.7 In Vitro RNA Sequencing and Differential Gene GO Annotation Analysis

RNA was extracted from the control group, the olaparib-treated group (5 μM, 48 h), the IFN-γ-treated group (100 ng/ml, 48 h), and the combination therapy group (48 h). The samples were sent to Shanghai Majorbio Bio-Pharm Technology Co., Ltd. for sequencing. Differentially expressed genes (DEGs) were identified using DESeq2 software, with the thresholds set at $|\log_2 FC| \geq 1.0$ and an adjusted p value (p_{adj}) < 0.05. Gene Ontology (GO) annotation analysis was performed on the DEGs utilizing the GO database, which categorizes genes based on their involvement in Biological Processes (BP), Cellular Components (CC), and Molecular Functions (MF). The gene set associated with the regulation of T cell activation was obtained from the Gene Ontology (GO) term Regulation of T cell activation. The oxidative stress-related gene set was derived from the publication [1].

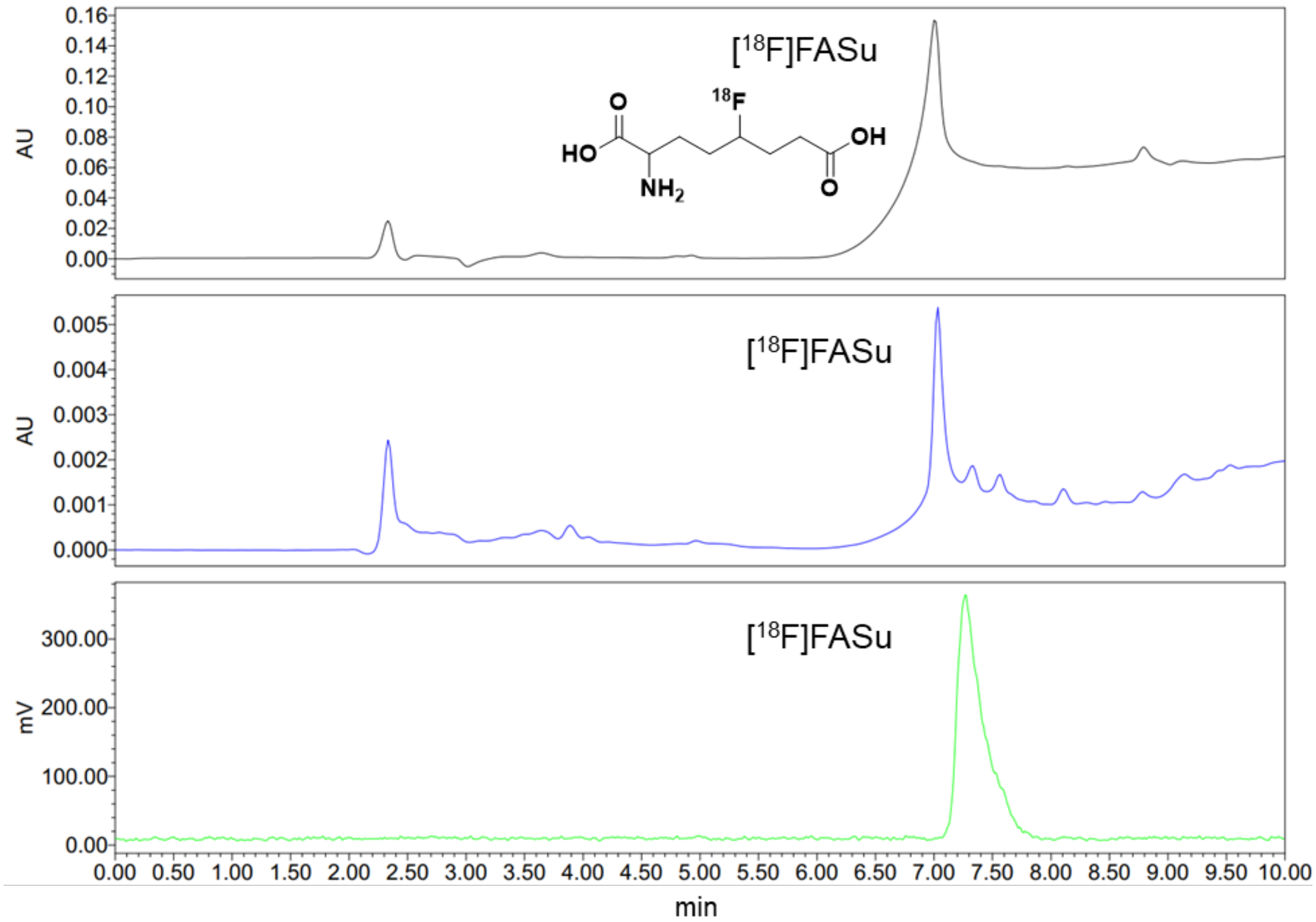

Supplementary Fig. 1. Characterization of [^{18}F]FASu. [^{18}F]FASu was characterized on reverse phase HPLC using Waters C18 column. UV 280 nm (top), UV 256 nm (middle), γ tracer (bottom).

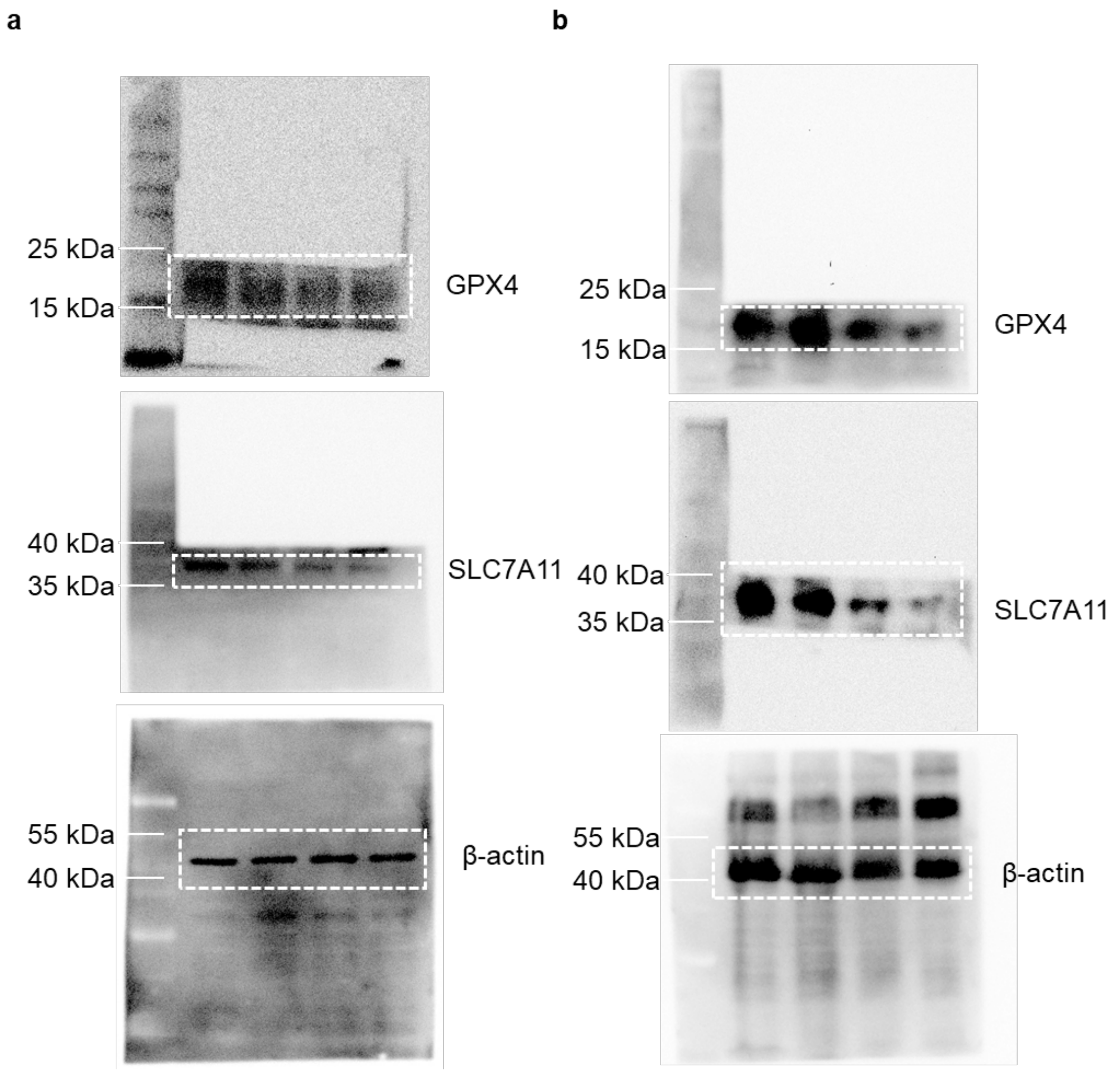

Supplementary Fig. 2. Uncropped Western blot images. Areas with high background were masked with aluminum foil during imaging.

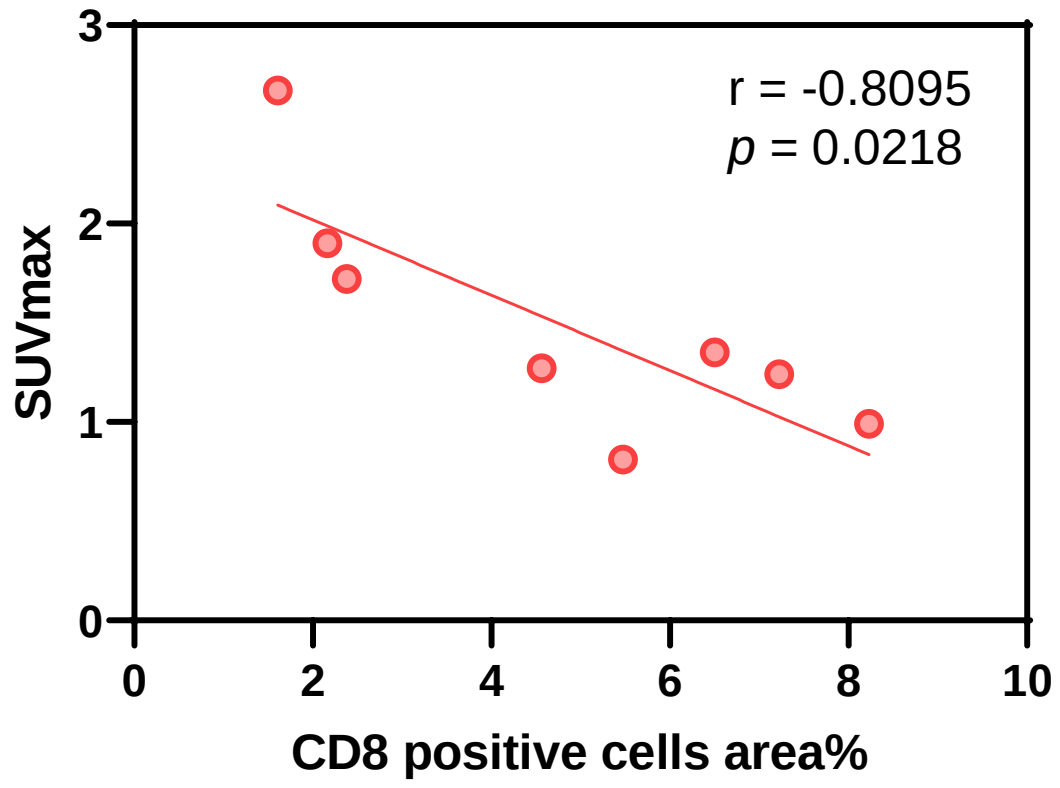

Supplementary Fig. 3. Analysis of the correlation between intratumoral $CD8^+$ expression and [^{18}F]FASu SUVmax at day 15, as determined by immunohistochemistry.

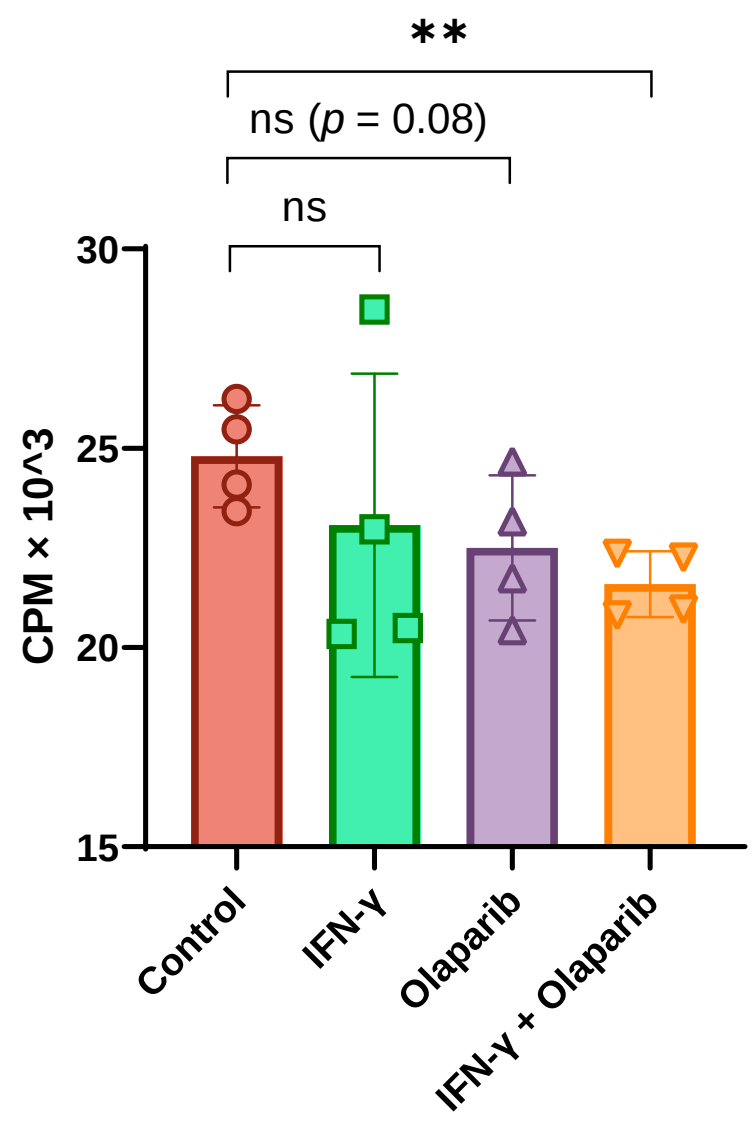

Supplementary Fig. 4. Cellular uptake assay. Hepa1-6 cells were treated for 16 hours with IFN-γ (100 ng/mL), Olaparib (1 μM), a combination of both, or an equivalent volume of DMSO as a control. After two washes with PBS, the cells were incubated with [^{18}F]FASu for 1 hour. Following incubation, the cells were quickly washed twice with ice-cold PBS and then lysed with 1 M NaOH for 1 minute. The resulting cell lysate was collected for further analysis. The CPM value was significantly lower in the combination therapy group than in the control group (Control group = 24802 ± 1282, IFN-γ group = 23071 ± 3806, Olaparib group = 22502 ± 1823, Combination group =21592 ± 825.5). CPM = Count Per Minute.

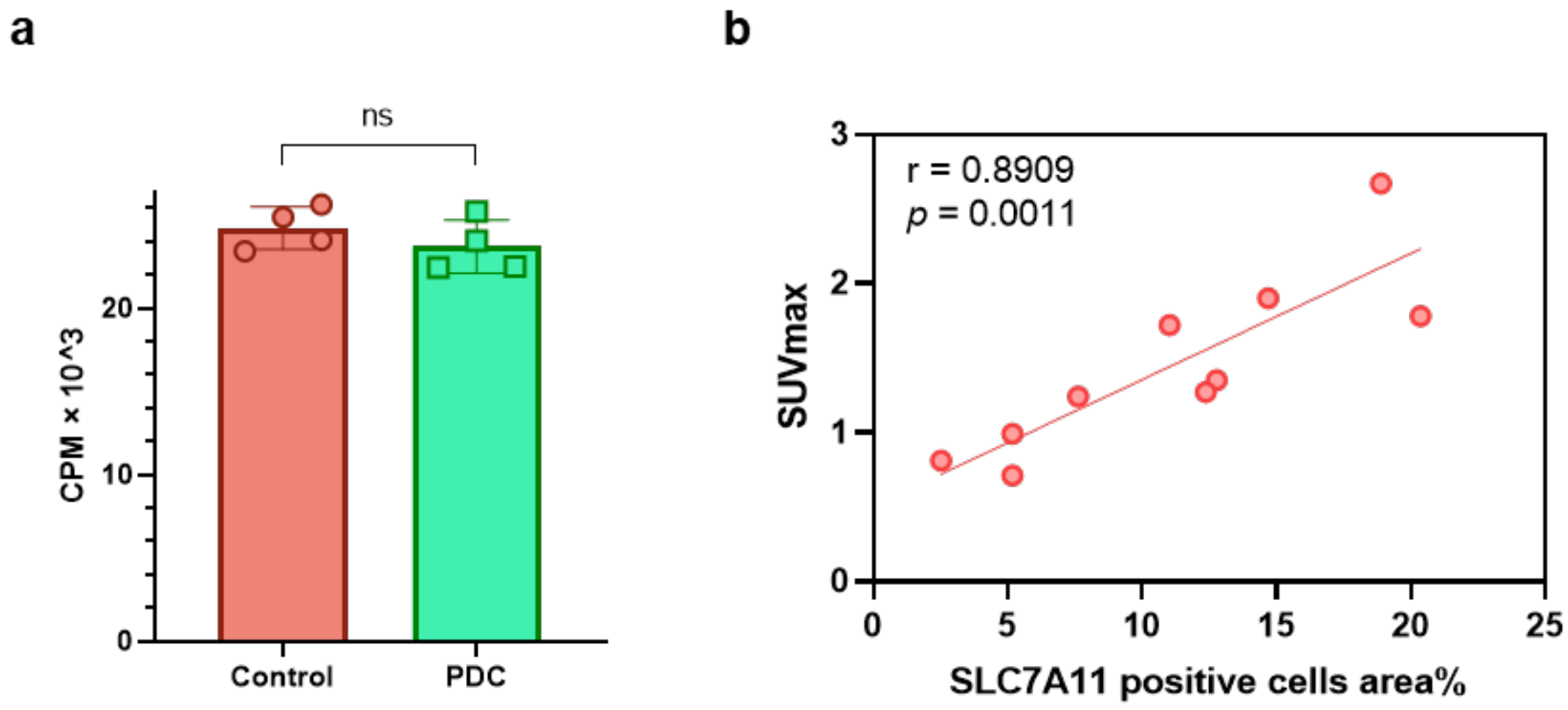

Supplementary Fig. 5. (a) The uptake of [^{18}F]FASu is not affected by EAAT inhibition. The hepa1-6 cells were coincubated with PDC at 1 mM for 16h. (b) Correlation of SLC7A11 protein expression detected by IHC with [^{18}F]FASu uptake (SUVmax) in C57BL/6J animal models. PDC = L-trans-pyrrolidine-2,4-dicarboxylic acid (an EAAT inhibitor). ns, not statistically significant.

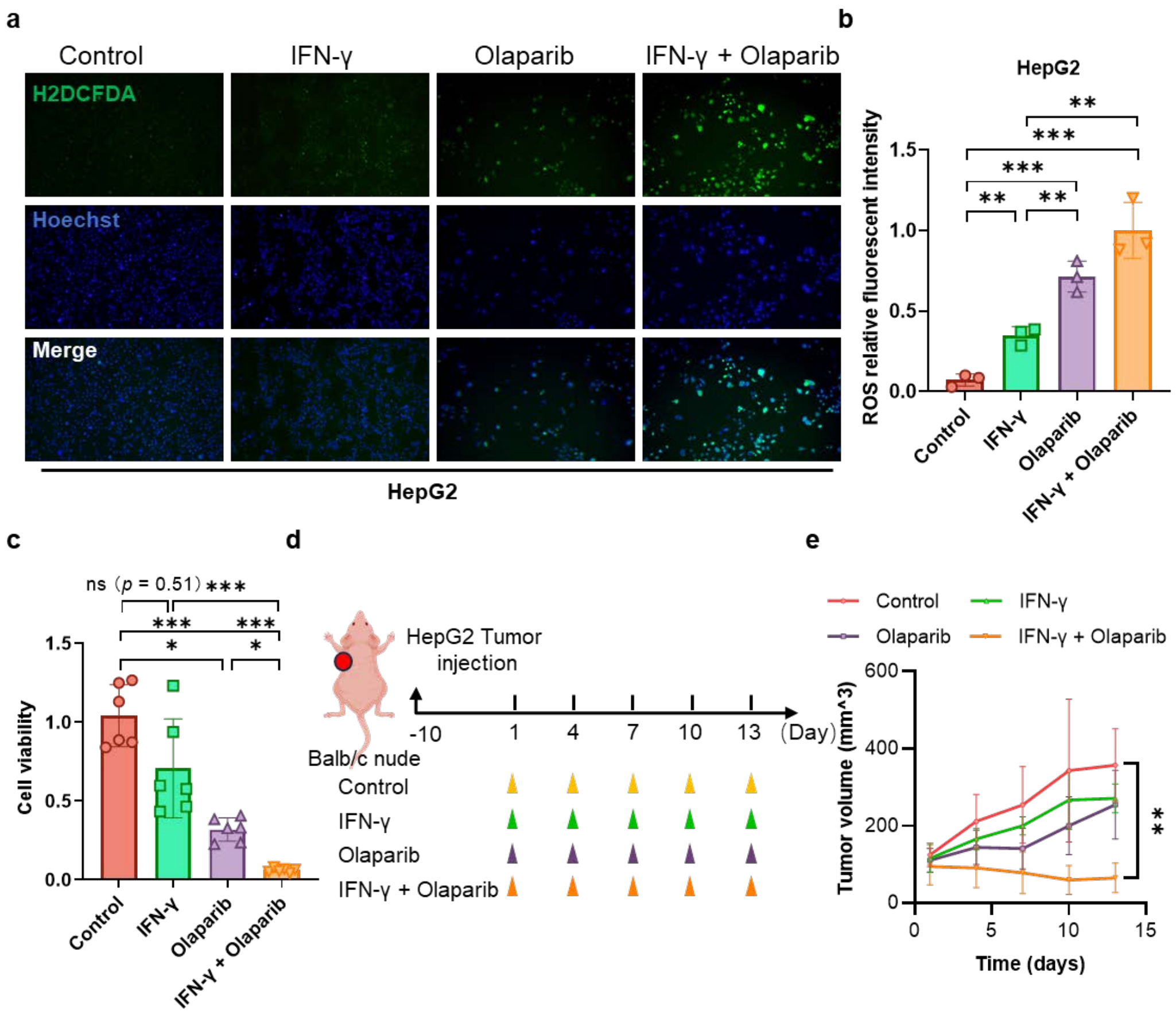

Supplementary Fig. 6. Combination treatment with IFN-γ and Olaparib is effective and increases ROS levels in HepG2 cells. (a) HepG2 cells were treated for 16 h with IFN-γ (100 ng/mL), Olaparib (1 μM), a combination of both, or a control (an equivalent volume of DMSO), followed by staining with the ROS-sensitive fluorescent probe H2DCFDA (5 μM) for 30 min; (b) The relative fluorescence intensity was significantly higher in the combination group than in the control and monotherapy groups (Control: 0.07 ± 0.04; IFN-γ: 0.35 ± 0.05; Olaparib: 0.71 ± 0.10; IFN-γ + Olaparib: 1.00 ± 0.17); (c) Following 16-hour treatment with the aforementioned drug concentrations using CCK-8 kit, cell viability analysis demonstrated marked cytotoxicity for the combination group (Control: 1.04 ± 0.20; IFN-γ: 0.71 ± 0.31; Olaparib: 0.32 ± 0.08; IFN-γ + Olaparib: 0.06 ± 0.01); (d-e) Effect of IFN-γ and Olaparib on HepG2 tumor growth in vivo. HepG2 cells (1×10^7 cells) were subcutaneously inoculated into BALB/c Nude mice. Mice were treated either with IFN-γ (1.25×10^5 U per mouse), Olaparib (100 mg/kg) or both. n ≥ 6 animals in each group. *$p < 0.05$, **$p < 0.01$, ***$p < 0.001$, ****$p < 0.0001$.

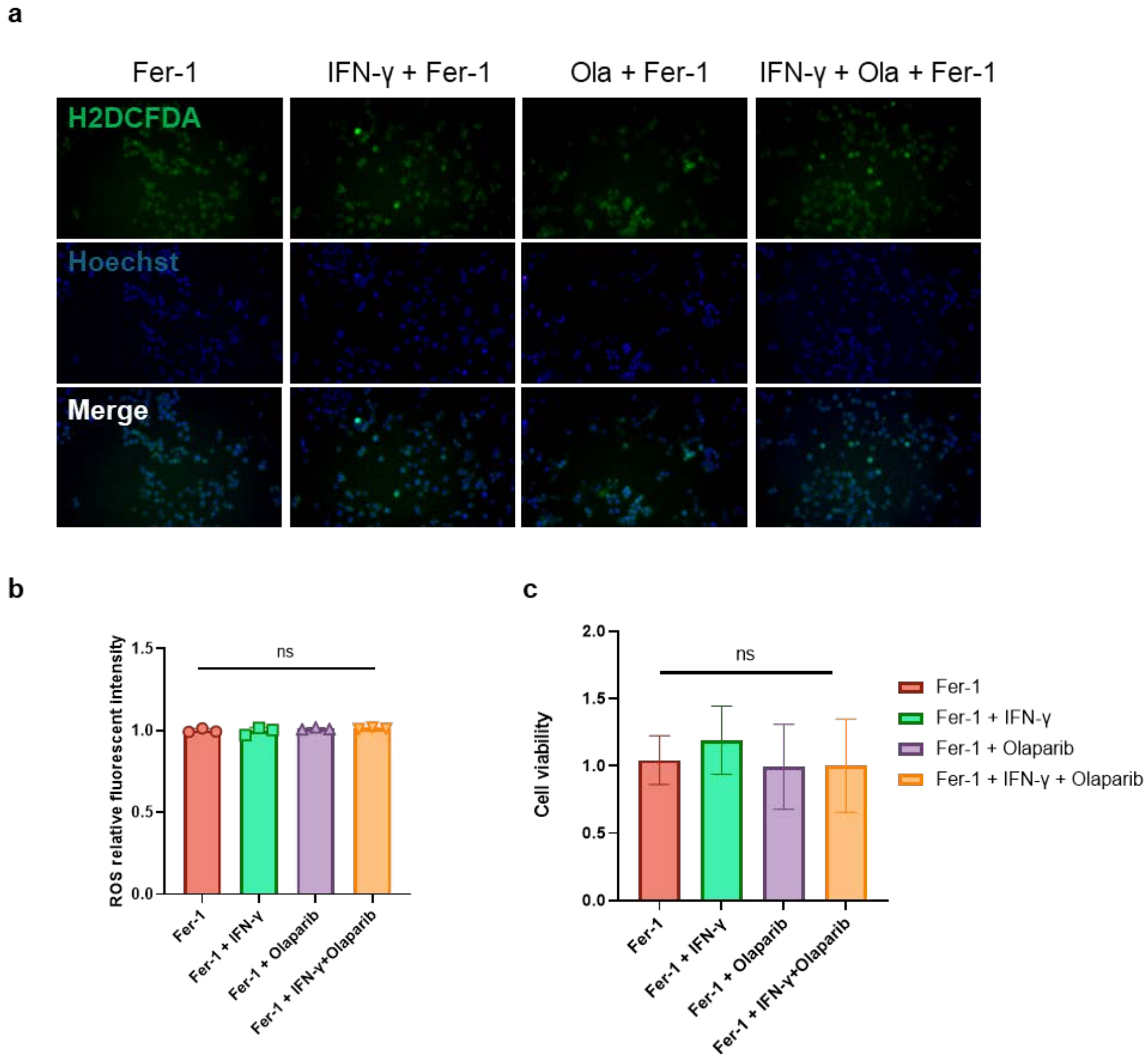

Supplementary Fig. 7. Ferrostatin-1 can reverse the therapeutic effect of the combination of Olaparib and IFN-γ. (a) The cells were first pretreated with 2 μM Fer-1 for 14 h, followed by co-incubation with various drugs for 24 h. After treatment, the cells were washed thrice with ice-cold PBS and subjected to H2DCFDA and Hoechst staining; (b) There was no statistically significant difference in relative fluorescence intensity among the different treatment groups; (c) Cell viability analysis demonstrated marked cytotoxicity for the combination group (Control: 1.04 ± 0.18; IFN-γ: 1.19 ± 0.25; Olaparib: 0.99 ± 0.32; IFN-γ + Olaparib: 1.00 ± 0.35, n = 5). ns, not statistically significant.

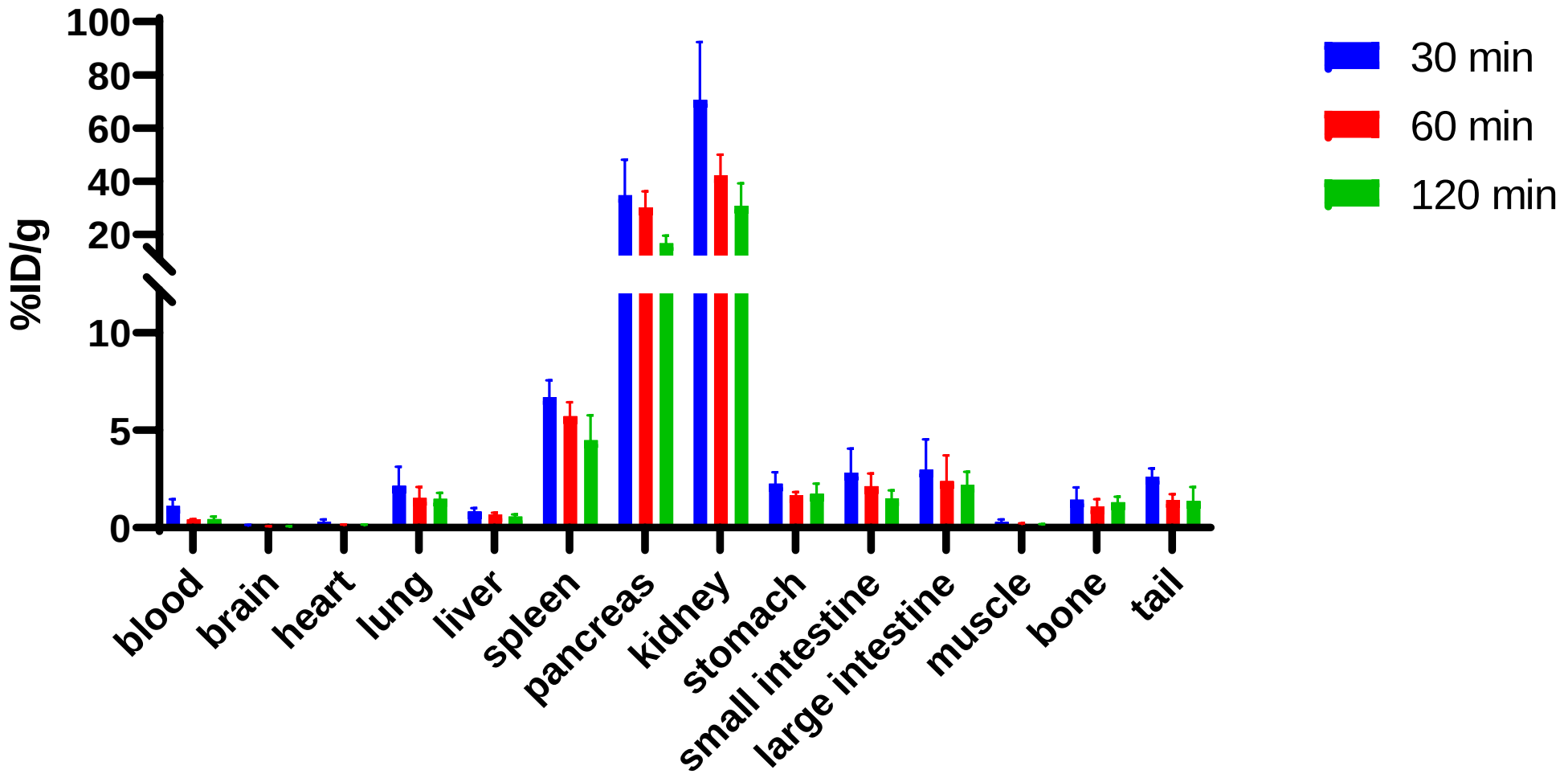

Supplementary Fig. 8. Biodistribution of [^{18}F]FASu at different time points after tail vein injection in C57BL/6J animal model (n = 4).

Supplementary Table 1

Therapeutic targets for immune checkpoint inhibitor monotherapy in HCC.

Treatment	Target 1	Target 2	Imaging Probe	Study Type	Reference
Pembrolizumab	PD-1	-	-	Clinical	[2,3]
Camrelizumab	PD-1	-	-	Clinical	[4]
Sintilimab	PD-1	-	-	Clinical	[5]
Nivolumab	PD-1	-	-	Clinical	[6]
Tremelimumab	CTLA-4	-	-	Clinical	[7,8]
Cemiplimab	PD-L1	-	-	Clinical	[9]
ICIs	PD-1	-	CT/MRI	Clinical	[10]
Nivolumab	PD-1	-	CT/MRI	Clinical	[11,12]
Pembrolizumab	PD-1	-	CT/MRI	Clinical	[13]
Tislelizumab	PD-1	-	CT/MRI	Clinical	[14]
Anti-PD-(L)1	PD-1/PD-L1	-	-	Clinical	[15]

Anti-PD-L1	PD-L1	-	-	Preclinical	[16]
ICIs	PD-1/PD-L1	-	-	Clinical	[17]
Anti-PD-1	PD-1	-	-	Preclinical	[18-21]
ICIs	PD-1/PD-L1		MRI	Clinical	[22]

Supplementary Table 2

Therapeutic targets for immune checkpoint inhibitor combination therapy in HCC.

Treatment	Target 1	Target 2	Imaging Probe	Study Type	Reference
Nivolumab + Ipilimumab	PD-1	CTLA-4	-	Clinical	[23,24]
Atezolizumab + Bevacizumab	PD-L1	VEGFA	-	Clinical/Preclinical	[25-50]
Sintilimab + Bevacizumab biosimilar	PD-1	VEGFA	-	Clinical	[20,51]
Lenvatinib + Pembrolizumab	PD-1	VEGFR	-	Clinical	[52]
Camrelizumab + Rivoceranib	PD-1	VEGFR	-	Clinical	[53]
Tremelimumab + Durvalumab	PD-L1	CTLA-4	[^{18}F]FDG PET/CT	Clinical	[54-58]
Camrelizumab + Apatinib	PD-1	VEGFR	-	Clinical	[59-61]
Atezolizumab + Cabozantinib	PD-L1	VEGFR	-	Clinical	[62]
TACE + ICIs + anti-VEGF /TKIs	PD-1/PD-L1	VEGFR	-	Clinical	[63]
AFP vaccine + ICIs	PD-1/PD-L1	AFP	-	Preclinical	[64]
Avelumab + Axitinib	PD-L1	VEGFR	-	Clinical	[65]

ICIs ± targeted therapy	PD-1/PD-L1	VEGFR/Multikinase	-	Clinical	[66]
ICIs + Lenvatinib	PD-1	VEGFR	CT/MRI	Clinical	[67]
Nivolumab/Pembrolizumab + TKI	PD-1/PD-L1	VEGFR/Multikinase	CT/MRI	Clinical	[68]
Ipilimumab + Nivolumab/Pembrolizumab	PD-1	CTLA-4	-	Clinical	[69]
ICIs + Anti-angiogenesis therapies	PD-L1/PD-1	VEGFR	US	Clinical	[70]
Camrelizumab + Lenvatinib	PD-1	VEGFR	-	Clinical	[71]
Envafolimab + Suvemcitug	PD-L1	VEGFR	-	Clinical	[72]
Anti-PD-L1 + Berberine	PD-L1	Erk signaling pathway	-	Preclinical	[73]
RT + Anti-PD-1 + Anti-VEGFR2	PD-1	VEGFR	-	Preclinical	[74]
ICIs + Bevacizumab/Lenvatinib	PD-1/PD-L1	VEGFR	-	Clinical	[75]
Anti-PD-1/PD-L1 + Kinase Inhibitors	PD-1/PD-L1	VEGFR	-	Clinical	[76]
ICIs+ Molecular Agents	PD-1/PD-L1	VEGFR	-	Clinical	[77]
ICIs + Lenvatinib/Regorafenib	PD-1	VEGFR	-	Clinical	[78]
ICIs + Lenvatinib/Bevacizumab	PD-1/PD-L1	VEGFR	-	Clinical	[79]
Anti-PD-1 + Lenvatinib	PD-1	VEGFR	-	Clinical	[80]
Anti-PD-L1 + Cordycepin	PD-L1	DNA and RNA	-	Preclinical	[81]

ICIs + TKI	PD-1/PD-L1	VEGFR	-	Clinical	[82]
Anti-PD-1 + Tc3	PD-1	PRDX1	-	Preclinical	[83]
Anti-PD-L1 + XAV939	PD-L1	Wnt pathway	-	Preclinical	[84]
ICIs + Ipilimumab	PD-1/PD-L1	CTLA-4	-	Clinical	[85]
ICIs + Angiogenesis inhibitors	PD-1/PD-L1	VEGFR	-	Clinical	[86]
Envafolimab + Lenvatinib	PD-L1	VEGFR	-	Clinical	[87]
ICIs+ TKI + RT + HAIC	PD-1	VEGFR	-	Clinical	[88]
Anti-PD-1/PD-L1 + TKI	PD-1/PD-L1	VEGFR	-	Clinical	[89]
Anti-PD-1 + TKI	PD-1	VEGFR	-	Clinical	[90]
Anti-PD-1 + OTX008	PD-1	Galectin-1	-	Preclinical	[91]
Anti-PD1 + Anti-VEGFR2	PD-1	VEGFR	US	Preclinical	[92]
Anti-PD-1 + LSD1 inhibitor	PD-1	LSD1		Preclinical	[93]
Tremelimumab + Durvalumab	PD-L1	CTLA-4	-	Clinical	[94]
Anti-PD-1 + MIT/NAP nanoformulation	STAT3	cGAS-STING pathway	-	Preclinical	[95]
Anti-PD-1 + Oleanolic acid	PD-1	-	-	Preclinical	[96]
Anti-PD-L1 + AZD5069	PD-L1	CXCR2	-	Preclinical /Clinical	[97]
Anti-PD-1 + TACE	PD-1	-	MRI	Clinical	[98]

Ropeginterferon alfa-2b + Nivolumab	PD-1	Interferon alpha receptor	-	Clinical	99
Anti-PD-1 + SBRT	PD-1	-	-	Preclinical	100
Anti-PD-1 + Lenvatinib	PD-1	VEGFR	-	Preclinical /Clinical	101,102
miR-107-3p + CD133 Ab-NBs	PD-L1	CD133	-	Preclinical	103
Anti-PD-1 + CB-5083	PD-1	VCP	-	Preclinical	104

[a] HCC = hepatocellular carcinoma; ICI = immune checkpoint inhibitor; TKI = tyrosine kinase inhibitors; CT = computed tomography; MRI = magnetic resonance imaging; PET = positron emission computed tomography; US = ultrasound; VCP = valosin-containing protein; HAIC = hepatic arterial infusion chemotherapy; SBRT = stereotactic body radiotherapy; TACE = transcatheter arterial chemoembolization; RT = radiation therapy.

[b] Certain drugs target multiple kinases. For instance, lenvatinib inhibits VEGFR1–3, PDGFR, FGFR1–4, and RET. In our table, only primary targets are retained.

Supplementary Table 3

Biodistribution of [^{18}F]FASu in Normal C57BL/6J Mice at Different Time Points Post-injection

	Time (min)		
Organ	30 (n = 4)	60 (n = 4)	120 (n = 4)
Blood	1.11 ± 0.35	0.41 ± 0.04	0.44 ± 0.13
Bone	1.43 ± 0.63	1.10 ± 0.37	1.30 ± 0.28
Brain	0.10 ± 0.04	0.07 ± 0.01	0.06 ± 0.02
Heart	0.31 ± 0.12	0.14 ± 0.01	0.12 ± 0.04
Kidney	70.53 ± 21.85	42.17 ± 7.73	30.86 ± 8.26
Large intestine	2.96 ± 1.58	2.38 ± 1.33	2.20 ± 0.67
Liver	0.83 ± 0.18	0.69 ± 0.08	0.58 ± 0.11
Lung	2.15 ± 0.98	1.52 ± 0.58	1.47 ± 0.31
Muscle	0.29 ± 0.12	0.18 ± 0.05	0.16 ± 0.02
Pancreas	34.68 ± 13.37	30.10 ± 6.00	16.66 ± 2.80
Small intestine	2.81 ± 1.23	2.12 ± 0.65	1.50 ± 0.40

Spleen	6.68 ± 0.88	5.71 ± 0.72	4.50 ± 1.26
Stomach	2.27 ± 0.57	1.64 ± 0.17	1.75 ± 0.50
Tail	2.61 ± 0.43	1.42 ± 0.30	1.37 ± 0.72

Values (%ID/g) are presented as mean ± SD.

References

1 Wang, H. *et al.* A four oxidative stress gene prognostic model and integrated immunity-analysis in pancreatic adenocarcinoma. *Front. Oncol.* **12**, 1015042 (2022).

2 Finn, R. S. *et al.* Pembrolizumab As Second-Line Therapy in Patients With Advanced Hepatocellular Carcinoma in KEYNOTE-240: A Randomized, Double-Blind, Phase III Trial. *J. Clin. Oncol.* **38**, 193-202 (2020).

3 Zhu, A. X. *et al.* Pembrolizumab in patients with advanced hepatocellular carcinoma previously treated with sorafenib (KEYNOTE-224): a non-randomised, open-label phase 2 trial. *Lancet Oncol.* **19**, 940-952 (2018).

4 Qin, S. *et al.* Camrelizumab in patients with previously treated advanced hepatocellular carcinoma: a multicentre, open-label, parallel-group, randomised, phase 2 trial. *Lancet Oncol.* **21**, 571-580 (2020).

5 Wang, K. *et al.* Adjuvant sintilimab in resected high-risk hepatocellular carcinoma: a randomized, controlled, phase 2 trial. *Nat. Med.* **30**, 708-715 (2024).

6 El-Khoueiry, A. B. *et al.* Nivolumab in patients with advanced hepatocellular carcinoma (CheckMate 040): an open-label, non-comparative, phase 1/2 dose escalation and expansion trial. *Lancet* **389**, 2492-2502 (2017).

7 Sangro, B. *et al.* A clinical trial of CTLA-4 blockade with tremelimumab in patients with hepatocellular carcinoma and chronic hepatitis C. *J. Hepatol.* **59**, 81-88 (2013).

8 Duffy, A. G. *et al.* Tremelimumab in combination with ablation in patients with advanced hepatocellular carcinoma. *J. Hepatol.* **66**, 545-551 (2017).

9 Freitas-Lopes, M. A., Mafra, K., David, B. A., Carvalho-Gontijo, R. & Menezes, G. B. Differential Location and Distribution of Hepatic Immune Cells. *Cells* **6**, 48 (2017).

10 Zhang, F. *et al.* Alpha-fetoprotein combined with initial tumor shape irregularity in predicting the survival of patients with advanced hepatocellular carcinoma treated with immune-checkpoint inhibitors: a retrospective multi-center cohort study. *J. Gastroenterol.* **60**, 442-455 (2025).

11 Yau, T. *et al.* Nivolumab versus sorafenib in advanced hepatocellular carcinoma (CheckMate 459): a randomised, multicentre, open-label, phase 3 trial. *Lancet Oncol.* **23**, 77-90 (2022).

12 Shu, P., Wang, F. & Wang, X. Case Report: Abscopal effect in a patient with refractory metastatic hepatocellular carcinoma treated with stereotactic body radiotherapy and PD-1 inhibitor. *Front. Immunol.* **16**, 1536689 (2025).

13 Merle, P. *et al.* Pembrolizumab as Second-Line Therapy for Advanced Hepatocellular Carcinoma: Longer Term Follow-Up from the Phase 3 KEYNOTE-240 Trial. *Liver Cancer* **12**, 309-320 (2023).

14 Qin, S. *et al.* Tislelizumab vs Sorafenib as First-Line Treatment for Unresectable Hepatocellular Carcinoma: A Phase 3 Randomized Clinical Trial. *JAMA Oncol.* **9**, 1651-1659 (2023).

15 Zhu, H. F. *et al.* Combination of alpha-fetoprotein and neutrophil-to-lymphocyte ratio to predict treatment response and survival outcomes of patients with unresectable hepatocellular carcinoma treated with immune checkpoint inhibitors. *BMC Cancer* **23**, 547 (2023).

16 Liang, J. *et al.* Anti-PD-L1 blockade facilitates antitumor effects of radiofrequency ablation by improving tumor immune microenvironment in hepatocellular carcinoma. *Apoptosis* **30**, 55-68 (2025).

17 Jiang, K. *et al.* Evaluation of surrogate endpoints in phase III randomized control trials of advanced hepatocellular carcinoma treated with immune checkpoint inhibitors. *Eur. J. Clin. Pharmacol.* **81**, 727-737 (2025).

18 Hirata, T. *et al.* Comparative Analysis of Systemic Immune Responses and Metastatic Risks in Tumor Ablation: An Animal Study of Radiofrequency Ablation and Irreversible Electroporation with Immune Modulation. *Cardiovasc. Intervent. Radiol.* **48**, 524-535 (2025).

19 Wang, P. *et al.* CXCL9 Overexpression Predicts Better HCC Response to Anti-PD-1 Therapy and Promotes N1 Polarization of Neutrophils. *J. Hepatocell. Carcinoma* **11**, 787-800 (2024).

20 Ren, Z. *et al.* Phase 2 study of serplulimab with the bevacizumab biosimilar HLX04 in the first-line treatment of advanced hepatocellular carcinoma. *Cancer Immunol. Immunother.* **74**, 69 (2025).

21 Xu, K. *et al.* ADH1C maintains the homeostasis of metabolic microenvironment to inhibit steatotic hepatocellular carcinoma. *Metabolism* **168**, 156267 (2025).

22 Aoki, T. *et al.* Higher Enhancement Intrahepatic Nodules on the Hepatobiliary Phase of Gd-EOB-DTPA-Enhanced MRI as a Poor Responsive Marker of Anti-PD-1/PD-L1 Monotherapy for Unresectable Hepatocellular Carcinoma. *Liver Cancer* **10**, 615-628 (2021).

23 Yau, T. *et al.* Efficacy and Safety of Nivolumab Plus Ipilimumab in Patients With Advanced Hepatocellular Carcinoma Previously Treated With Sorafenib: The

CheckMate 040 Randomized Clinical Trial. *JAMA Oncol.* **6**, e204564 (2020).
24 Kaseb, A. O. *et al.* Perioperative nivolumab monotherapy versus nivolumab plus ipilimumab in resectable hepatocellular carcinoma: a randomised, open-label, phase 2 trial. *Lancet Gastroenterol. Hepatol.* **7**, 208-218 (2022).
25 Finn, R. S. *et al.* Atezolizumab plus Bevacizumab in Unresectable Hepatocellular Carcinoma. *N. Engl. J. Med.* **382**, 1894-1905 (2020).
26 Chuma, M. *et al.* Clinical significance of circulating biomarkers of immune-checkpoint molecules with atezolizumab plus bevacizumab therapy in unresectable hepatocellular carcinoma. *Hepatol. Int.* **18**, 1472-1485 (2024).
27 Ohama, H. *et al.* Clinical usefulness of newly developed prognostic predictive score for atezolizumab plus bevacizumab for hepatocellular carcinoma. *Cancer Rep. (Hoboken)* **7**, e2042 (2024).
28 Ueno, M. *et al.* CRAFITY score as a predictive marker for refractoriness to atezolizumab plus bevacizumab therapy in hepatocellular carcinoma: a multicenter retrospective study. *J. Gastroenterol.* **59**, 1107-1118 (2024).
29 Hatanaka, T. *et al.* Development and validation of a modified albumin-bilirubin grade and alpha-fetoprotein score (mALF score) for hepatocellular carcinoma patients receiving atezolizumab and bevacizumab. *Hepatol. Int.* **17**, 86-96 (2023).
30 Sasaki, R. *et al.* Evaluating the Role of Hepatobiliary Phase of Gadoxetic Acid-Enhanced Magnetic Resonance Imaging in Predicting Treatment Impact of Lenvatinib and Atezolizumab plus Bevacizumab on Unresectable Hepatocellular Carcinoma. *Cancers (Basel)* **14**, 827 (2022).
31 Rossari, F. *et al.* alpha-FAtE: A new predictive score of response to atezolizumab plus bevacizumab for unresectable hepatocellular carcinoma. *Int. J. Cancer* **154**, 1043-1056 (2024).
32 Yang, H. *et al.* High serum IL-6 correlates with reduced clinical benefit of atezolizumab and bevacizumab in unresectable hepatocellular carcinoma. *JHEP Rep.* **5**, 100672 (2023).
33 Myojin, Y. *et al.* Interleukin-6 Is a Circulating Prognostic Biomarker for Hepatocellular Carcinoma Patients Treated with Combined Immunotherapy. *Cancers (Basel)* **14**, 883 (2022).
34 Inukai, Y. *et al.* Intestinal Microbiome Associated with Efficacy of Atezolizumab and Bevacizumab Therapy for Hepatocellular Carcinoma. *Cancers (Basel)* **16**, 1675 (2024).
35 Giovannini, C. *et al.* Low-Baseline PD1+ Granulocytes Predict Responses to Atezolizumab-Bevacizumab in Hepatocellular Carcinoma. *Cancers (Basel)* **15**, 1661 (2023).
36 Murai, H. *et al.* Multiomics identifies the link between intratumor steatosis and the exhausted tumor immune microenvironment in hepatocellular carcinoma. *Hepatology* **77**, 77-91 (2023).

37 Wu, Y. L. *et al.* Neutrophil-to-Lymphocyte and Platelet-to-Lymphocyte Ratios as Prognostic Biomarkers in Unresectable Hepatocellular Carcinoma Treated with Atezolizumab plus Bevacizumab. *Cancers (Basel)* **14**, 5834 (2022).

38 Hosoda, S. *et al.* Low Baseline CXCL9 Predicts Early Progressive Disease in Unresectable HCC with Atezolizumab Plus Bevacizumab Treatment. *Liver Cancer* **12**, 156-170 (2023).

39 Shirane, Y. *et al.* Peripheral T Cell Subpopulations as a Potential Surrogate Biomarker during Atezolizumab plus Bevacizumab Treatment for Hepatocellular Carcinoma. *Cancers (Basel)* **16**, 1328 (2024).

40 Gramantieri, L. *et al.* Circulating CD8 lymphocytes predict response to atezolizumab-bevacizumab in hepatocellular carcinoma. *Eur. J. Immunol.* **54**, e2350637 (2024).

41 Jost-Brinkmann, F. *et al.* Atezolizumab plus bevacizumab in unresectable hepatocellular carcinoma: Results from a German real-world cohort. *Aliment. Pharmacol. Ther.* **57**, 1313-1325 (2023).

42 Ishihara, N. *et al.* Association between tumor morphology and efficacy of atezolizumab plus bevacizumab for advanced hepatocellular carcinoma. *Hepatol. Res.* **54**, 773-780 (2024).

43 Qin, S. *et al.* Atezolizumab plus bevacizumab versus active surveillance in patients with resected or ablated high-risk hepatocellular carcinoma (IMbrave050): a randomised, open-label, multicentre, phase 3 trial. *Lancet* **402**, 1835-1847 (2023).

44 Cheng, A. L. *et al.* Updated efficacy and safety data from IMbrave150: Atezolizumab plus bevacizumab vs. sorafenib for unresectable hepatocellular carcinoma. *J. Hepatol.* **76**, 862-873 (2022).

45 Suzuki, T. *et al.* Serum interleukin-6 levels at the start of the second course of atezolizumab plus bevacizumab therapy predict therapeutic efficacy in patients with advanced hepatocellular carcinoma: a multicenter analysis. *J. Gastroenterol. Hepatol.* **39**, 2158-2168 (2024).

46 Hatanaka, T. *et al.* Prognostic impact of C-reactive protein and alpha-fetoprotein in immunotherapy score in hepatocellular carcinoma patients treated with atezolizumab plus bevacizumab: a multicenter retrospective study. *Hepatol. Int.* **16**, 1150-1160 (2022).

47 Ochi, H. *et al.* Usefulness of neutrophil-to-lymphocyte ratio in predicting progression and survival outcomes after atezolizumab-bevacizumab treatment for hepatocellular carcinoma. *Hepatol. Res.* **53**, 61-71 (2023).

48 Vithayathil, M. *et al.* Machine learning based radiomic models outperform clinical biomarkers in predicting outcomes after immunotherapy for hepatocellular carcinoma. *J. Hepatol.* **83**, 959-970 (2025).

49 Lehrich, B. M. *et al.* Precision targeting of beta-catenin induces tumor reprogramming and immunity in hepatocellular cancers. *Nat. Commun.* **16**, 5009 (2025).

50 Chuang, C. H., Chen, C. T., Hsu, C. H. & Shao, Y. Y. Carry-over effect of immunotherapy in patients with advanced hepatocellular carcinoma. *Cancer Immunol. Immunother.* **74**, 208 (2025).

51 Ren, Z. *et al.* Sintilimab plus a bevacizumab biosimilar (IBI305) versus sorafenib in unresectable hepatocellular carcinoma (ORIENT-32): a randomised, open-label, phase 2-3 study. *Lancet Oncol.* **22**, 977-990 (2021).

52 Llovet, J. M. *et al.* Lenvatinib plus pembrolizumab versus lenvatinib plus placebo for advanced hepatocellular carcinoma (LEAP-002): a randomised, double-blind, phase 3 trial. *Lancet Oncol.* **24**, 1399-1410 (2023).

53 Qin, S. *et al.* Camrelizumab plus rivoceranib versus sorafenib as first-line therapy for unresectable hepatocellular carcinoma (CARES-310): a randomised, open-label, international phase 3 study. *Lancet* **402**, 1133-1146 (2023).

54 Abou-Alfa, G. K. *et al.* Plain language summary of the HIMALAYA study: tremelimumab and durvalumab for unresectable hepatocellular carcinoma (liver cancer). *Future Oncol.* **19**, 2505-2516 (2023).

55 Song, X. *et al.* Exposure-Response Analyses of Tremelimumab Monotherapy or in Combination with Durvalumab in Patients with Unresectable Hepatocellular Carcinoma. *Clin. Cancer Res.* **29**, 754-763 (2023).

56 Chamseddine, S. *et al.* Plasma Growth Hormone as a Prognostic Biomarker to Durvalumab and Tremelimumab in Patients with Advanced Hepatocellular Carcinoma. *J. Hepatocell. Carcinoma* **11**, 455-461 (2024).

57 Lim, K. *et al.* Population Pharmacokinetics and Exposure-Response Analysis of Tremelimumab 300 mg Single Dose Combined with Durvalumab 1500 mg Q4W (STRIDE) in Patients with Unresectable Hepatocellular Carcinoma. *J. Clin. Pharmacol.* **63**, 1221-1231 (2023).

58 Abou-Alfa, G. K. *et al.* Tremelimumab plus Durvalumab in Unresectable Hepatocellular Carcinoma. *NEJM Evid.* **1**, EVIDoa2100070 (2022).

59 Xia, Y. *et al.* Efficacy and safety of camrelizumab plus apatinib during the perioperative period in resectable hepatocellular carcinoma: a single-arm, open label, phase II clinical trial. *J. Immunother. Cancer* **10**, e004656 (2022).

60 Zhang, T. Q. *et al.* Camrelizumab (a PD-1 inhibitor) plus apatinib (an VEGFR-2 inhibitor) and hepatic artery infusion chemotherapy for hepatocellular carcinoma in Barcelona Clinic Liver Cancer stage C (TRIPLET): a phase II study. *Signal Transduct. Target. Ther.* **8**, 413 (2023).

61 Doherty, D. G. & O'Farrelly, C. Innate and adaptive lymphoid cells in the human liver. *Immunol. Rev.* **174**, 5-20 (2000).

62 Kelley, R. K. *et al.* Cabozantinib plus atezolizumab versus sorafenib for advanced hepatocellular carcinoma (COSMIC-312): a multicentre, open-label, randomised, phase 3 trial. *Lancet Oncol.* **23**, 995-1008 (2022).

63 Jin, Z. C. *et al.* Immune checkpoint inhibitors and anti-vascular endothelial growth factor antibody/tyrosine kinase inhibitors with or without transarterial chemoembolization as first-line treatment for advanced hepatocellular carcinoma (CHANCE2201): a target trial emulation study. *EClinicalMedicine* **72**, 102622 (2024).

64 Lu, X. *et al.* Combination of AFP vaccine and immune checkpoint inhibitors slows hepatocellular carcinoma progression in preclinical models. *J. Clin. Invest.* **133**, e163291 (2023).

65 Kudo, M. *et al.* Avelumab in Combination with Axitinib as First-Line Treatment in Patients with Advanced Hepatocellular Carcinoma: Results from the Phase 1b VEGF Liver 100 Trial. *Liver Cancer* **10**, 249-259 (2021).

66 Zhu, C. *et al.* Characterizations of multi-kingdom gut microbiota in immune checkpoint inhibitor-treated hepatocellular carcinoma. *J. Immunother. Cancer* **12**, e008686 (2024).

67 Guo, D. Z. *et al.* Circulating immune index predicting the prognosis of patients with hepatocellular carcinoma treated with lenvatinib and immunotherapy. *Front. Oncol.* **13**, 1109742 (2023).

68 Lee, P. C. *et al.* Gut microbiota and metabolites associate with outcomes of immune checkpoint inhibitor-treated unresectable hepatocellular carcinoma. *J. Immunother. Cancer* **10**, e004779 (2022).

69 Wong, J. S. L. *et al.* Ipilimumab and nivolumab/pembrolizumab in advanced hepatocellular carcinoma refractory to prior immune checkpoint inhibitors. *J. Immunother. Cancer* **9**, e001945 (2021).

70 Zhang, Y. *et al.* Quantitative Parameters of Contrast-Enhanced Ultrasound Predicting the Response to Combined Immune Checkpoint Inhibitor and Anti-angiogenesis Therapies for Unresectable Hepatocellular Carcinoma. *Ultrasound Med. Biol.* **50**, 352-357 (2024).

71 Zhang, S. *et al.* The use of peripheral CD3(+)gammadelta(+)Vdelta2(+) T lymphocyte cells in combination with the ALBI score to predict immunotherapy response in patients with advanced hepatocellular carcinoma: a retrospective study. *J. Cancer Res. Clin. Oncol.* **150**, 365 (2024).

72 Ma, L. *et al.* Anti-PD-L1 envafolimab combined with anti-VEGF suvemcitug in pretreated solid tumors and hepatocellular carcinoma: an open-label phase II study with safety run-in stage. *Invest. New Drugs* **43**, 181-190 (2025).

73 Miao, G. *et al.* Berberine in combination with anti-PD-L1 suppresses hepatocellular carcinoma progression and metastasis via Erk signaling pathway. *Ann. Med. Surg. (Lond.)* **87**, 103-112 (2025).

74 Sheng, H. *et al.* Antiangiogenic Treatment Facilitates the Abscopal Effect of Radiation Therapy Combined With Anti-PD-1 in Hepatocellular Carcinoma. *Int. J. Radiat. Oncol. Biol. Phys.* **121**, 534-546 (2025).

75 Cui, X. *et al.* Characteristics and outcomes of primary and secondary resistance to immune checkpoint inhibitors in hepatocellular carcinoma. *Cancer Immunol. Immunother.* **74**, 239 (2025).

76 Lu, Y. & Lu, Y. Clinical predictive factors of the efficacy of immune checkpoint inhibitors and kinase inhibitors in advanced hepatocellular cancer. *Clin. Transl. Oncol.* **27**, 1142-1154 (2025).

77 Zhong, S. *et al.* Combining immune checkpoint inhibitors and molecular-targeted agents with hepatic arterial infusion chemotherapy for hepatocellular carcinoma with inferior vena cava and/or right atrium tumor thrombus. *Hepatol. Int.* **19**, 560-575 (2025).

78 Yu, Z. *et al.* Comparative Efficacy of Lenvatinib Plus Immunotherapy and Regorafenib Plus Immunotherapy After Lenvatinib Failure for Advanced Hepatocellular Carcinoma: A Retrospective Study. *Drugs Real World Outcomes* **12**, 135-143 (2025).

79 Pan, Z. *et al.* Comparison of Efficacy between Lenvatinib and Bevacizumab in Combination of Immune Checkpoint Inhibitor and Interventional Triple Therapy in Chinese Advanced Hepatocellular Carcinoma: The CLEAP 2302 Study. *Liver Cancer* **14**, 601-619 (2025).

80 Guo, X. *et al.* Contrasting cytotoxic and regulatory T cell responses underlying distinct clinical outcomes to anti-PD-1 plus lenvatinib therapy in cancer. *Cancer Cell* **43**, 248-268 e249 (2025).

81 Liang, B. G. *et al.* Cordycepin mediates pyroptosis in HCC through the upregulation of TXNIP and synergizes with anti-PD-L1 immunotherapy. *Hepatol. Commun.* **9**, e0633 (2025).

82 Lu, M. *et al.* Diagnostic Model for Proliferative HCC Using LI-RADS: Assessing Therapeutic Outcomes in Hepatectomy and TKI-ICI Combination. *J. Magn. Reson. Imaging* **61**, 134-147 (2025).

83 Hu, X. *et al.* Discovery of indole analogue Tc3 as a potent pyroptosis inducer and identification of its combination strategy against hepatic carcinoma. *Theranostics* **15**, 1285-1303 (2025).

84 Shao, Y. Y. *et al.* Downregulation of PD-L1 expression by Wnt pathway inhibition to enhance PD-1 blockade efficacy in hepatocellular carcinoma. *Biol. Direct* **20**, 49 (2025).

85 Zheng, S. S. *et al.* Efficacy and Safety of Ipilimumab Plus Anti-PD-1/PD-L1 Antibodies Combination Therapy in Advanced Hepatocellular Carcinoma Patients Progressing After Multiple Lines of Treatment: A Retrospective Multicenter Study. *J. Hepatocell. Carcinoma* **12**, 527-537 (2025).

86 Xu, J., Wang, X., Jia, Z. & Sun, G. Effectiveness and safety of angiogenesis inhibitors combined with PD-1/PD-L1 blockades in the first-line treatment of patients with advanced hepatocellular carcinoma: A single-center retrospective study. *Medicine (Baltimore)* **104**, e41814 (2025).

87 Jiang, Y. *et al.* Efficacy and safety of the combination of envafolimab and lenvatinib in unresectable hepatocellular carcinoma: a single-arm, multicentre, exploratory phase II clinical study. *Invest. New Drugs* **43**, 18-29 (2025).

88 Tan, H. Y. *et al.* Efficacy of radiotherapy combined with hepatic arterial infusion chemotherapy, TKI and ICI for hepatocellular carcinoma with portal vein tumor thrombus: a retrospective cohort study. *Abdom. Radiol. (NY)* **50**, 1320-1329 (2025).

89 Pan, H. *et al.* Immune checkpoint inhibitor plus tyrosine kinase inhibitor with or without transarterial chemoembolization for unresectable hepatocellular carcinoma. *Front. Oncol.* **15**, 1385304 (2025).

90 Shen, Y., Xu, Y., Teng, Y., Ding, X. & Chen, J. First-line treatment of hepatocellular carcinoma: a propensity-matched analysis of tyrosine kinase inhibitors combined with TACE, with or without PD-1 inhibitors. *Front. Pharmacol.* **16**, 1533471 (2025).

91 Yu, X. *et al.* Galectin-1-Induced Tumor Associated Macrophages Repress Antitumor Immunity in Hepatocellular Carcinoma Through Recruitment of Tregs. *Adv. Sci. (Weinh)* **12**, e2408788 (2025).

92 Liu, X. *et al.* Inhibiting B-cell-mediated Immunosuppression to Enhance the Immunotherapy Efficacy in Hepatocellular Carcinoma. Preprint at https://doi.org/10.21203/rs.3.rs-6355345/v1 (2025).

93 Liang, C. *et al.* Lysine-specific demethylase 1 deletion reshapes tumour microenvironment to overcome acquired resistance to anti-programmed death 1 therapy in liver cancer. *Clin. Transl. Med.* **15**, e70335 (2025).

94 Myojin, Y. *et al.* Multiomics analysis of immune correlatives in hepatocellular carcinoma patients treated with tremelimumab plus durvalumab. *Gut* **74**, 983-995 (2025).

95 Wang, L. *et al.* Napabucasin deactivates STAT3 and promotes mitoxantrone-mediated cGAS-STING activation for hepatocellular carcinoma chemo-immunotherapy. *Biomaterials* **313**, 122766 (2025).

96 Tu, X. *et al.* Oleanolic acid inhibits M2 macrophage polarization and potentiates anti-PD-1 therapy in hepatocellular carcinoma by targeting miR-130b-3p-PTEN-PI3K-Akt signaling and glycolysis. *Phytomedicine* **141**, 156750 (2025).

97 Kwong, T. T. *et al.* Overcoming immunotherapy resistance in hepatocellular carcinoma by targeting myeloid IL-8/CXCR2 signaling. *Mol. Ther.* **33**, 1659-1673 (2025).

98 Gu, J. *et al.* Prediction of PD-1 Expression and Outcomes of Combined Therapy in Hepatocellular Carcinoma: an MRI-Based Radiomics Approach. *J. Imaging Inform. Med.* **39**, 299-310 (2026).

99 Qin, A., Ho, M. C., Tsai, C. Y., Liu, C. J. & Chen, P. J. Sequential combination with ropeginterferon alfa-2b and anti-PD-1 treatment as adjuvant therapy in HBV-related HCC: a phase 1 dose escalation trial. *Hepatol. Int.* **19**, 547-559 (2025).

100 Xu, K. *et al.* Stereotactic body radiation therapy (SBRT) increases anti-PD-1 antitumor activity by enhancing the tumor immune microenvironment in mice with metastatic hepatocellular carcinoma. *Discov. Oncol.* **16**, 1081 (2025).

101 Pan, G. Q. *et al.* Targeting SAMD1 enhances the effect of anti-PD-1 plus lenvatinib therapy in hepatocellular carcinoma by increasing ferroptosis sensitivity and immune response. *Metabolism* **170**, 156304 (2025).

102 Jiang, J. *et al.* The immune-reinforcements of Lenvatinib plus anti-PD-1 and their rationale to unite with TACE for unresectable hepatocellular carcinoma treatment. *Immunol. Lett.* **275**, 107003 (2025).

103 Zhang, C. *et al.* Ultrasound nanobubble-based combinational strategies of loaded miR-107-3p and CD133 Ab for anti-PD-L1 and anti-hepatocellular cancer stem cells. *Int. J. Pharm.* **670**, 125140 (2025).

104 Cheng, C. *et al.* VCP downstream metabolite glycerol-3-phosphate (G3P) inhibits CD8(+)T cells function in the HCC microenvironment. *Signal Transduct. Target. Ther.* **10**, 26 (2025).
